# Cryptic Kultarr: Integrative Taxonomy Reveals Unrecognized Species of Carnivorous Marsupial (Dasyuridae: *Antechinomys*) in Arid Australia

**DOI:** 10.1002/ece3.71618

**Published:** 2025-06-26

**Authors:** Cameron S. Dodd, Renee A. Catullo, Kenny J. Travouillon, Andrew M. Baker, Michael Westerman, Linette S. Umbrello

**Affiliations:** ^1^ School of Biological Sciences University of Western Australia Perth Western Australia Australia; ^2^ Collections and Research Western Australian Museum Perth Western Australia Australia; ^3^ School of Biology and Environmental Science Queensland University of Technology Brisbane Queensland Australia; ^4^ Biodiversity and Geosciences Program Queensland Museum South Brisbane Queensland Australia; ^5^ Department of Environment and Genetics La Trobe University Melbourne Victoria Australia

**Keywords:** arid zone, cryptic species, gibber plain, morphology, phylogenetics, Sminthopsinae, systematics

## Abstract

Globally, mammal species are experiencing unprecedented rates of extinction. Despite this, many small mammals remain understudied and even undescribed, posing a major barrier to effective conservation planning. Without formal study and documentation, it is impossible to make well‐informed management decisions for these species. This issue is particularly pronounced in Australia, where not only is the mammal extinction rate the highest in the world, but the vast inaccessibility of much of the continent, combined with a shortage of expert taxonomists, has left a large portion of small mammal diversity poorly documented. One example of this is the kultarr ( *Antechinomys laniger* ), a small, insectivorous marsupial in the family Dasyuridae that is distributed across much of the Australian arid zone. While currently considered a single species, previous morphological and molecular studies have suggested the presence of cryptic taxa within the group. Here, we resolve the taxonomy of the kultarr using 12S mitochondrial sequence data and genome‐wide single nucleotide polymorphisms. We identify three clades of kultarr that are morphologically distinct, largely allopatric, and show minimal evidence of genetic admixture. These clades differ in hindfoot length, body size, ear size, and craniodental morphology. The clades consist of an eastern semi‐arid clade, a central Channel Country/Kati Thanda‐Lake Eyre Basin clade, and a western deserts clade. We rediagnose *A. laniger* sensu stricto as the eastern clade, resurrect *A. spenceri* as the central clade, and describe a new species, *A. auritus* sp. nov., as the western clade. This research highlights the importance of integrating morphological and genetic data in small mammal taxonomy and emphasizes the need for comprehensive geographic sampling within widespread species complexes.

## Graphical abstract

Conservation is impossible if we have not described all of the species we aim to conserve. This paper reviews the taxonomy of the kultarr ( *Antechinomys laniger* ) a small arid‐zone specialist native to Australia. We describe a new species, *A. auritus* sp. nov., and resurrect a formerly recognized species, *A. spenceri* .

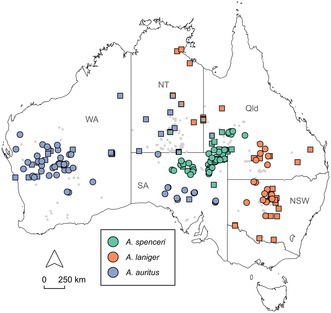

## 1 Introduction

Current global extinction rates may be as high as 1000 times the predicted background extinction rate (Pimm et al. 2014), largely due to the direct and indirect effects of human activity (Bull and Maron 2016). Among terrestrial vertebrates, mammals have experienced the highest extinction rates (Pimm et al. 2014), with Australia having the highest mammal extinction rate worldwide. More than 10% of the ~300 known pre‐European species are now extinct (Woinarski et al. 2015), accounting for one‐third of all global modern mammal extinctions despite Australia representing only ~5% of the global mammal species diversity (Davidson et al. 2017). Importantly, these figures only consider species formally described by Western Science, meaning the true extinction count is likely higher (Jackson et al. 2022). Indeed, 18 modern Australian mammal species have been described post‐extinction from museum material to date (e.g., Mahoney et al. 2007; Travouillon et al. 2019).

One group with particularly high cryptic diversity and taxonomic uncertainty is the marsupial family Dasyuridae. Since 2000, modern genetic methods have facilitated the description of 18 new dasyurid taxa (AMTC 2024), yet substantial diversity remains unresolved (Blacket et al. 1999, 2000; Westerman et al. 2023). One such example is the kultarr ( *Antechinomys laniger* ), a small, insectivorous marsupial that superficially resembles a jerboa (Dipodidae) or a hopping mouse (*Notomys* spp.) due to their elongated limbs and tail. Currently, all populations of kultarr are treated as a single species ( *A. laniger* ), although morphological (Archer 1977; Lidicker and Marlow 1970) and genetic (Westerman et al. 2023) evidence suggests that cryptic species may exist in this group. The kultarr is not listed as threatened under the federal E*nvironment Protection and Biodiversity Conservation Act 1999* (DCCEEW 2024), although it is listed as Endangered in New South Wales under the *Biodiversity Conservation Act 2016* (NSW DPIE 2024). Conservation planning rarely accounts for undescribed species, making taxonomic reviews like this study essential to ensuring cryptic species receive appropriate protection and management.

The kultarr was first described by Western Science as *Phascogale lanigera* Gould 1856 based on a specimen collected from central New South Wales by T. Mitchell (Gould 1856). It was assigned to its own genus, *Antechinomys*, in 1867 (Krefft 1867) and almost 40 years later, a second species, *Antechinomys spenceri* Thomas 1906, was described based on two specimens collected from Charlotte Waters, Northern Territory by P. Byrne in 1896 (Spencer 1896; Thomas 1906). A taxonomic review by Lidicker and Marlow (1970) supported the recognition of two species of kultarr. However, Archer (1977) argued that the morphological overlap was too extensive to justify more than one species and synonymized both forms under *A. laniger* . However, Archer (1977) did note several unusual kultarr specimens that could represent novel taxa but emphasized that additional data would be needed to confirm this.

Kultarr taxonomy then remained stable until Westerman et al. (2023) used mitochondrial and nuclear markers to test for population structure across the species range. Their study found strong support for three clades within the kultarr: an eastern Queensland + New South Wales clade, corresponding to *A. laniger* as described by Lidicker and Marlow (1970); a central South Australia + Northern Territory clade; and a Western Australia clade (Westerman et al. 2023). Additionally, their results found strong evidence for the eastern + central clade positioned as sister to the western clade, contradicting Lidicker and Marlow's (1970) hypothesis that an eastern semi‐arid adapted clade ( *A. laniger* ) was sister to a central‐western arid‐adapted clade ( *A. spenceri* ).

**FIGURE 1:**
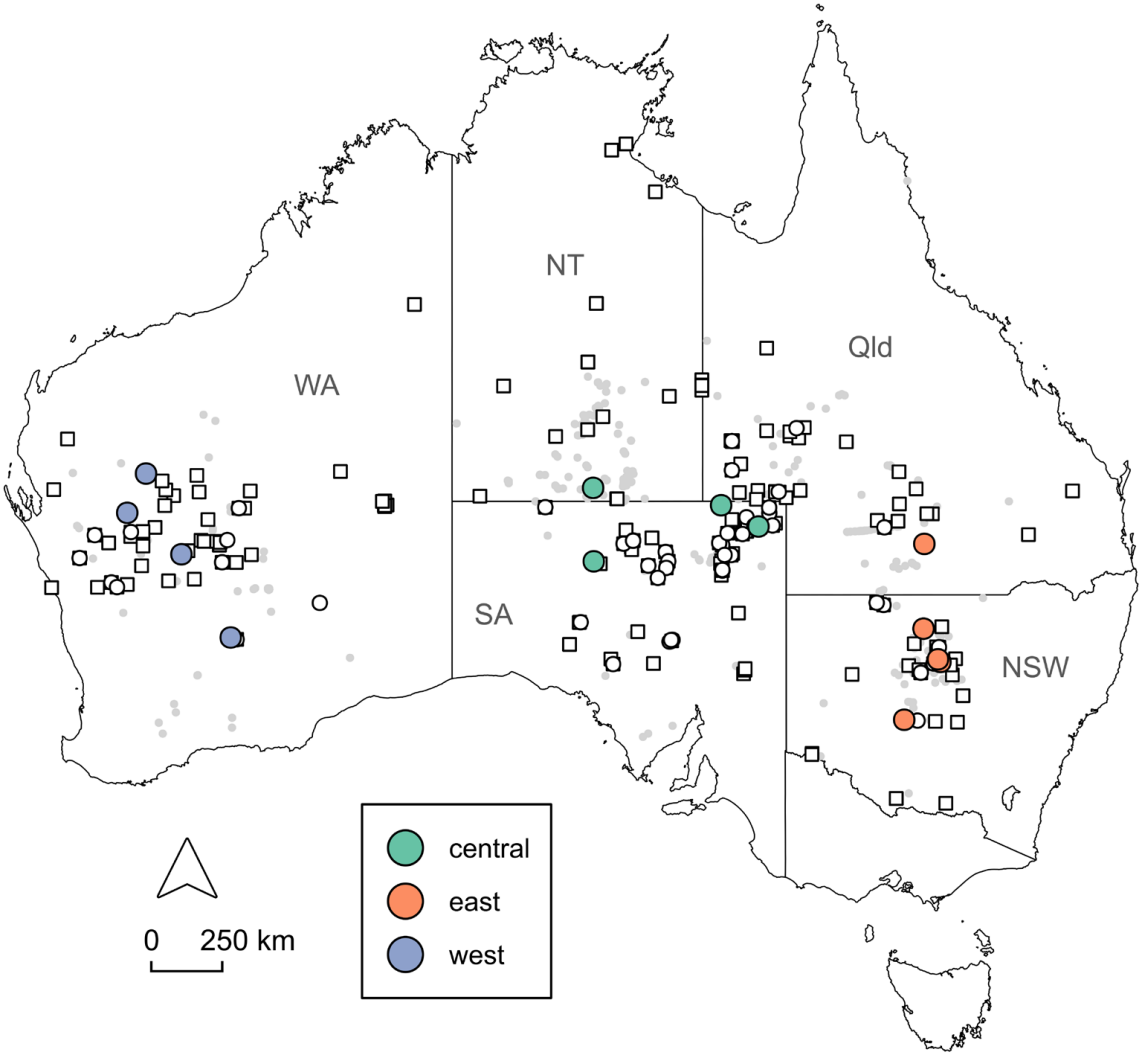
Map of known kultarr occurrence records (grey points), specimens examined as part of the present study (white squares), genetic data available for this study (white circles), and specimens previously identified as belonging to three distinct clades of kultarr (coloured circles) (Westerman et al. 2023), see legend. Map shows Australia with state and territory borders, those where kultarr have been recorded are labelled: NSW, New South Wales; NT, Northern Territory; Qld, Queensland; SA, South Australia; WA, Western Australia.

While Westerman et al. (2023) used robust phylogenetic methods, the sample size was limited (sequences from 14 specimens), and many areas of the kultarr's distribution were not sampled. Here, we reevaluate the taxonomic status of the clades identified by Westerman et al. (2023) using additional 12S mitochondrial sequences, genome‐wide nuclear single nucleotide polymorphisms (SNPs) and substantially expanded geographic sampling across Australia (Figure 1). Additionally, we analyse craniodental and whole‐body morphological data to assess morphological differences within and between clades. Finally, we use an integrative taxonomic approach (Padial et al. 2010) to revise the taxonomy of the kultarr.

## 2 Materials and Methods

### 2.1 Terminology

Historically, the genus name *Antechinomys* referred only to the kultarr ( *A. laniger* ). However, in 2023, the long‐tailed dunnart (previously *Sminthopsis longicaudata* ) was reclassified as *A. longicaudatus* (Westerman et al. 2023) after multiple phylogenetic analyses consistently recovered it as sister to *A. laniger* (Krajewski et al. 2012; Westerman et al. 2016, 2023). In this study, we use the term “*Antechinomys*” to refer to the entire genus ( *A. laniger* complex and *A. longicaudatus* ) and the term “kultarr” to specifically refer to the *A. laniger* complex, excluding *A. longicaudatus* .

### 2.2 Tissue Sample Processing and DNA Extraction

We obtained 68 modern kultarr tissue samples from the Western Australian Museum (WAM), Australian Museum (AMS), Queensland Museum (QM) and Australian Biological Tissue Collection (ABTC) at the South Australian Museum (SAMA) (Table A1 in Appendix 1). Four long‐tailed dunnart (*Antechinomys longicaudatus*) tissue samples were also obtained from the WAM for use as outgroups in phylogenetic analyses (Krajewski et al. 2012; Westerman et al. 2016, 2023). Modern tissues were stored at −80°C and comprised liver, muscle, or ear tissue.

Because some parts of the kultarr distribution were not represented by modern tissue samples, we collected skin samples from three historic museum‐prepared skins using the protocol described by Roycroft et al. (2022) to improve geographic coverage of samples (Table A1 in Appendix 1; Figure 1). We extracted DNA from modern tissue using the Qiagen DNeasy Tissue and Blood Kit according to the standard manufacturer's instructions. For historic skin samples, we used a modified Qiagen protocol based on Joseph et al. (2016). This protocol involved rehydrating tissue in 1 mL of ultrapure water for 6–8 h before placing the rehydrated sample into the lysis buffer and adding 40 μL of *proteinase K* during the overnight digestion step, rather than the standard 20 μL. All extractions were eluted twice (60 μL each) to maximize DNA yield as well as DNA concentration. We quantified elution concentration using a Qubit v.3.0 fluorometer and assessed DNA degradation via gel electrophoresis on a 1.5% agarose Invitrogen E‐Gel. All sample preparation and extractions were conducted in the Genetic Resources lab at the WAM in Perth, Australia.

### 2.3 Mitochondrial Sequence Data Generation

We amplified the mitochondrial 12S rRNA gene via polymerase chain reaction (PCR) in 25 μL volumes containing 2 μL of sample DNA extraction, 0.2 μM of both forward and reverse primers, 0.8 units of MyTAQ DNA polymerase (Applied Biosystems, Branchburg, NJ, USA) and 1 unit of PCR buffer containing 1.5 mM MgCl_2_ (Applied Biosystems). For historic skin samples, we used 3 μL of DNA template and added 0.03 mg of bovine serum albumin (BSA) (Invitrogen, Waltham, MA, USA) to improve PCR success (Kreader 1996). Amplification success was assessed by gel electrophoresis on a 1.5% agarose Invitrogen E‐Gel. We amplified the entire 970 bp sequence of 12S using the forward primer 12C (5′‐AAAGCAAAACACTGAAAATG‐3′) and reverse primer 12GG (5′‐TRGGTGTARGCTRRRTGCTTT‐3′) (Springer et al. 1995). Due to degradation of DNA from historic skin samples, we instead amplified shorter fragments using the forward primer 12C with two reverse primers: H12DV (5′‐TTAAACACACTTACGCCGTT‐3′) (Krajewski et al. 1997) yielding a 304 bp sequence; and H12S (5′‐TTACAGAACAGGCTCCTCTAG‐3′) (Springer et al. 1995), yielding a 565 bp sequence.

PCR amplification of modern samples followed Umbrello et al. (2024), as described in Krajewski et al. (1997). For historic samples, we used a simplified cycling protocol involving an initial denaturation step of 5 min at 95°C; an amplification step of: 95°C for 30 s, 45°C for 45 s, 72°C for 45 s, repeated 40 times; before a final holding step of 72°C for 10 min. All PCR products were sequenced at the Australian Genome Research Facility (AGRF, Perth, Australia). In total, the sequenced 12S dataset consisted of 38 sequences: 31 complete sequences (21 from this study and 10 from Genbank), six partial sequences (five from this study and one from Genbank), and one *A. longicaudatus* sequence from Genbank as an outgroup for phylogenetic analysis. The 12S sequences were aligned using Clustal Omega (v.1.2) (Sievers et al. 2011) as implemented in Geneious Prime (v.2024.0.4) (Kearse et al. 2012). The alignment was then manually inspected for ambiguously aligned sites which were excluded from the phylogenetic analyses.

### 2.4 Nuclear SNP Loci Generation

We sent a total of 95 samples (of extracted DNA) from 80 individuals to AGRF for double‐digest restriction‐associated DNA (ddRAD) sequencing using their genotyping by sequencing service. This included 48 high quality kultarr samples (including 15 as technical replicates), 28 low quality kultarr samples showing some evidence of degradation, and 4 *A. longicaudatus* samples as an outgroup. AGRF selected the restriction enzymes EcoRI and MspI after optimization. We processed raw sequence data on the *Stacks2* (Rochette et al. 2019) bioinformatic pipeline as implemented using *Galaxy* (v.24.1.3.dev0) (The Galaxy Community 2024). Further filtering and analyses were carried out in *R* (v.2024.04.2) (R Core Team 2023).

Sequence data were demultiplexed using *process_radtags*. Demultiplexed reads were then trimmed and filtered for read quality using Trimmomatric (v.0.36) (Bolger et al. 2014) using the parameters: *ILLUMINACLIP:TruSeq3:2:30:10; SLIDINGWINDOW:4:20; MINLEN:68; CROP:68*. We constructed de novo loci using *ustacks* with the following parameters: *m:6; M:2; max_locus_stacks:4*. A catalogue of loci was then generated using *cstacks* with default parameters using a subset of 24 individuals chosen to represent all mitochondrial clades (Schmidt et al. 2021; Westerman et al. 2023). Using default parameters, we matched reads from each sample to the catalogue using *sstacks* before transposing the data using *tsv2bam*. Finally, we ran *gstacks* and *populations* using default parameters on four subsets of samples (Table A1 in Appendix 1) to generate four datasets for different downstream analyses.

The first dataset (Phylogenetics Dataset) included all kultarr and long‐tailed dunnart samples and was optimized for high individual sample retention to enable phylogenetic tree construction. The second dataset (Population Genetics Dataset) included all kultarr samples and was optimized for high loci completeness to enable structure‐like analyses that identify the most likely number of populations (*K*) within kultarr. Two further datasets were generated containing samples from the two presumed contact zones identified by Westerman et al. (2023): the Central Admixture Dataset (south‐central deserts and stony plains) and the Eastern Admixture Dataset (central Queensland and New South Wales) (Figure 1). We exported all four datasets to R using the package *SNPfiltR* (v1.0.1) (DeRaad 2022) and applied additional filtering using *SNPfiltR* and *dartR.base* (v.0.65) (Gruber et al. 2018; Mijangos et al. 2022). Table A2 in Appendix 1 details downstream filtering steps and parameter settings for each pathway, while Table A3 in Appendix 1 provides sample subsets used in each analysis. The filtered datasets are summarized in Table 1.

**TABLE 1:** Summary statistics of the four SNP datasets used in the analysis of this study.

	Phylogenetics Dataset	Population Genetics Dataset	Central Admixture Dataset	East Admixture Dataset
# samples	70	62	17	13
# SNPs	5036	4463	1761	1392
Mean missing data	15.20%	5.53%	0%	0%

*Note:* Filter pipelines are detailed in the text and Table A2 in Appendix 1.

### 2.5 Phylogenetic Analyses

The final mitochondrial 12S alignment included 37 kultarr ( *Antechinomys laniger* ) samples and one long‐tailed dunnart ( *A. longicaudatus* ) as the outgroup. We generated a maximum likelihood (ML) phylogenetic tree using IQ‐TREE v.1.6.12 (Nguyen et al. 2015), with 1000 ultrafast bootstrap replicates (Hoang et al. 2018). Nucleotide substitution models were selected using ModelFinder (Kalyaanamoorthy et al. 2017) as implemented in IQ‐TREE. We separately fit substitution models for stem and loop regions of the 12S gene using a partition model (Chernomor et al. 2016) to account for differences in the evolutionary behavior of these two regions (Springer and Douzery 1996). Stem and loop regions were manually identified based on the 3D structure of the functional 12S transcript (Springer and Douzery 1996) following Westerman et al. (2023). ModelFinder identified a K2P + I model (Kimura 1980) and a TN + F model (Tamura and Nei 1993) as the best fit for the stem and loop regions respectively. The 12S gene was examined for diagnostic fixed differences between the clades identified by Westerman et al. (2023) following Singhal et al. (2018). Representative 12S sequences from each clade were then aligned to the phylogenetically closest mitochondrial genome available ( *Sminthopsis crassicaudata* , Genbank accession: NC_007631) to provide reference numbers for these sites.

For the SNP data, following filtering in R, the Phylogenetics Dataset, containing data from all kultarr and long‐tailed dunnart samples, was converted to a .*phylo* format using vcf2phylip (v.2.0) (Ortiz 2019). We then generated an ML phylogenetic tree using IQ‐TREE v.1.6.12 (Nguyen et al. 2015) with 1000 ultrafast bootstrap replicates (Hoang et al. 2018). We selected the best‐fit substitution model using ModelFinder (Kalyaanamoorthy et al. 2017), incorporating an ascertainment bias correction (Lewis 2001). ModelFinder identified a TVM + F + ASC + G4 model as the best fit for the SNP dataset.

### 2.6 Analyses of Population Structure and Admixture

To estimate the likely number of ancestral populations of kultarr, we calculated ancestry coefficients for *K* (number of populations) from *K* = 1 to *K* = 5 using the *snmf*() in the R package LEA (v.3.14.0) (Frichot and François 2015). For this, we used the Population Genetics Dataset, which contained only high‐quality samples, as ancestry coefficients can be sensitive to high levels of missing data (Gain and François 2021). We ran *snmf*() with three tolerance levels (0.0001, 0.00001 and 0.000001) and six regulation parameter (alpha) values (0, 1, 10, 100, 1000, 10,000) giving a total of 18 parameter combinations. All runs produced the same patterns of entropy changes between *K* values. The final model parameters were selected using the combination that yielded the lowest cross‐entropy values (tolerance = 0.0001 and alpha = 1000).

To assess admixture between populations, we calculated ancestry coefficients using *snmf*() on the two filtered SNP datasets containing only the samples from the presumed admixture zones as identified in the previous analysis (Central Admixture Dataset and Eastern Admixture Dataset). As each dataset contained two populations, we calculated ancestry coefficients for *K* = 2 in each instance. We restricted admixture analyses to individuals in the presumed admixture zones to prevent artificial inflation of admixture levels due to allele frequency variation across the broader population distribution. The Central Admixture Dataset consisted of individuals from the western clade collected from South Australia plus individuals from the central clades collected west of Kati Thanda‐Lake Eyre (longitude < 137°). The Eastern Admixture Dataset contained all seven individuals available from the eastern clade, along with six of the most easterly individuals from the central clade.

### 2.7 Morphological Data

**FIGURE 2:**
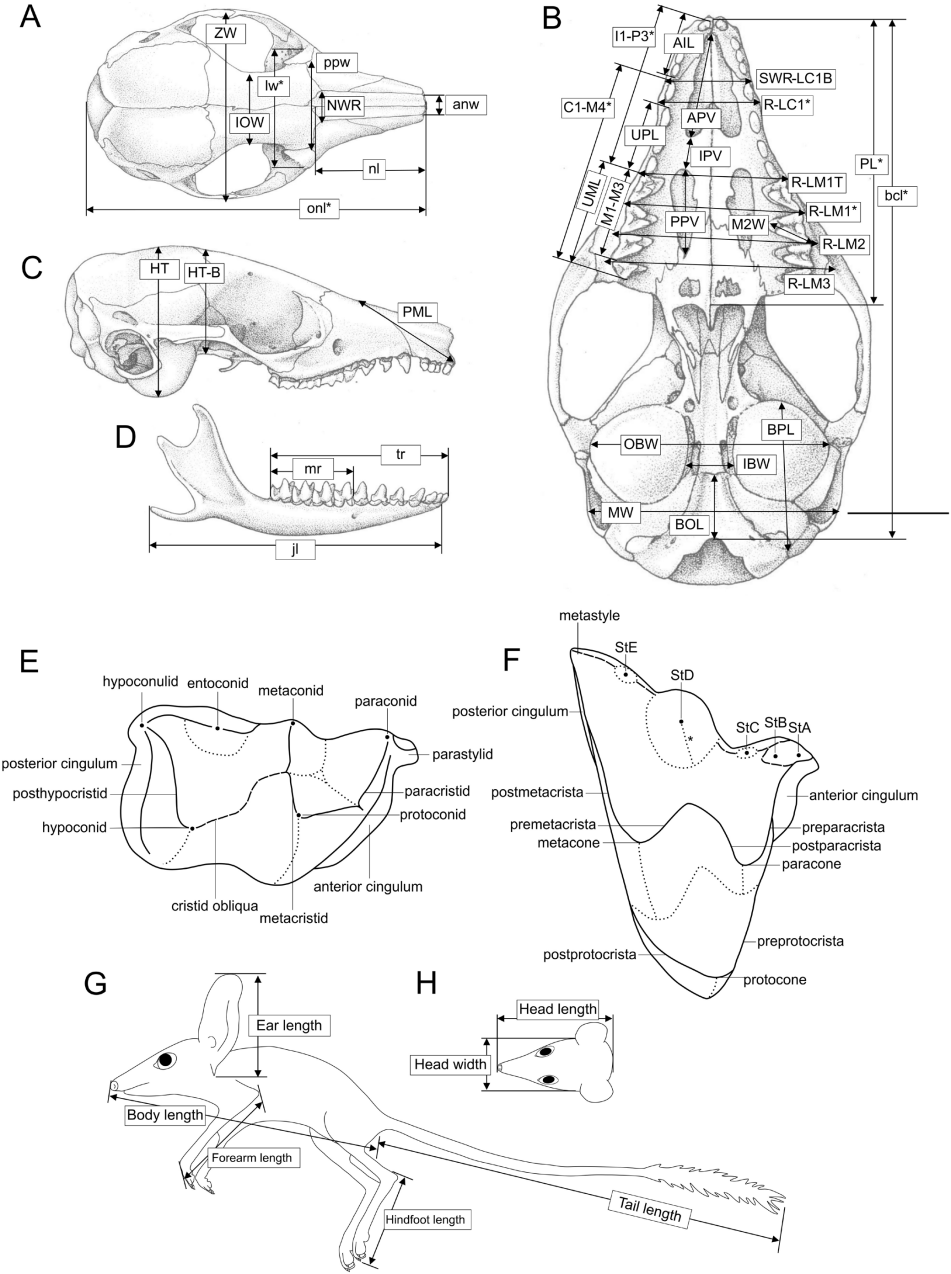
Linear measurements taken of kultarr ( *Antechinomys laniger* ) skulls (A–D) and whole bodies (G, H) as well as key structures of the upper (F) and lower (E) molars. (A) Dorsal view of cranium. (B) Ventral view of cranium. (C) Lateral view of cranium. (D) Lateral view of dentary. (E) Dorsobuccal view of a typical lower molar. (F) Ventrolingual view of a typical upper molar. (G) Lateral view of whole kultarr body. (H) Dorsal view of kultarr head. Character abbreviations and additional details of measurements are shown in Table 2. Kultarr skull diagrams adapted from Archer (1977) and molar diagrams adapted from Archer (1981). Whole body diagrams based on specimen WAM M5775.

We examined all available kultarr specimens from the WAM, SAMA, AMS, QM, and NMV (Table A1 in Appendix 1). We adapted craniodental and external body linear measurements from previous taxonomic studies of small marsupials (Archer 1977; Baker et al. 2015; Lidicker and Marlow 1970; Travouillon 2016) (Figure 2, Table 2). To minimize the effects of allometric and isometric growth, we only measured adult skulls (cranium and dentary) and bodies (Mosimann 1970). Adult skulls were identified by the absence of the upper and lower deciduous third premolars (dp3 and dP3) and complete eruption of both the adult third premolars (p3 and P3) and the fourth molars (m4 and M4) (Archer 1981). We identified spirit‐preserved specimens as adults by observing the dentition as above and/or by visually assessing sexual maturity, that is, full development of the testes (males) or pouch (females) (Woolley 1984). All measurements were made using Mitutoyo brand digital calipers to the nearest 0.1 mm. Specimens from localities where only one genetic clade was identified were assigned to the respective east, central, or west clades. For the small number of samples from areas where no genetic data was available (primarily in the Northern Territory), the morphology of genetically or locality‐identified specimens was used to assign clade identity.

**TABLE 2:** Craniodental characters measured in this study based on similar studies of small mammal taxonomy (Archer 1977; Baker et al. 2015; Lidicker and Marlow 1970; Travouillon 2016).

Abb.	Character	Description
AIL	Upper incisor length	Maximum length of upper incisor row
I1‐P3	I1 to P3 length	Length from anterior edge of I1 to posterior edge of P3
C1‐M4	C1 to M4 length	Length from anterior edge of C1 crown to posterior edge of M4 crown
UML	Upper molar length	Maximum length of upper molar row
M1‐M3	M1 to M3 length	Length from anterobuccal tip of M1 crown to posterobuccal tip of M3 crown
PML	Premaxilla length	Maximum length of premaxilla
APV	Anterior palatal vacuity length	Maximum length of anterior palatal vacuity
IPV	Inter‐palatal vacuity distance	Distance between anterior and posterior palatal vacuities
PPV	Posterior palatal vacuity length	Maximum length of posterior palatal vacuity
SWR‐LC1B	Width of palate anterior to C1	
R‐LC1	Width of palate posterior to C1	
R‐LM1T	Width of palate between P3 and M1	
R‐LM1	Width of palate between M1 and M2	
M2W	M2 width	Width of M2 from antero‐lingual edge of talonid to posterobuccal edge of crown
R‐LM2	Width of palate between M2 and M3	
R‐LM3	Width of palate between M3 and M4	
PL	Palate length	Length from anterior tip of premaxilla to posterior tip of the palatine
bcl	Basicranial length	Length from anterior tip of premaxilla to most anterior point of the foramen magnum
BPL	Bulla‐petrosal length	Length from anterior tip of bulla to posterior tip of petrosal
IBW	Inner bullar width	Width between bullae anterior to where the petrosal meets the lingual edge of the bullae
BOL	Basioccipital length	Length from centre of ventral basioccipital‐basisphenoid suture to the most anterior edge of the foramen magnum
MW	Squamosal width	Width of the squamosal directly posterior to the ectotympanic
OBW	Outer bullar width	Width of bullae at their widest point
tr	Lower toothrow length	Maximum length of lower toothrow
mr	Lower molar row length	Maximum length of lower molar row
jl	Jaw length	Maximum length of dentary
HT	Maximum skull height	Skull height at bullae
HT‐B	Maximal skull height minus bullae	Skull height directly anterior to the bullae
PML	Upper premolar length	Maximum length of upper premolar row
onl	Occipital‐nasal length	Length from anterior tip of nasal to the most posterior point of the occipital
ZW	Zygomatic width	Skull width at the widest point of the zygomatic arches
nl	Nasal length	Maximum nasal length
IOW	Inter‐orbital width	Frontal width at narrowest point between orbitals
lw	Lacrimal width	Skull width at the lacrimal foramina
ppw	Maximum width across postorbital ridge	Width of anterior edge of frontal between maxilla‐frontal‐lacrimal junctures
NWR	Posterior nasal width	Width of the nasal between the two nasal‐maxilla‐frontal junctures
anw	Anterior nasal width	Width of the nasal at its most anterior point

*Note:* Diagram of characters shown in Figure 2.

To visualize multidimensional craniodental shape variation, we conducted a principal component analysis (PCA). A subset of characters was chosen to ensure that no character correlated > 0.9 with any other to prevent artificially inflating the variance of certain skull regions (Büyüköztürk and Çokluk Bökeoğlu 2008) (Figure 2). PCA is sensitive to missing data, so we removed craniodental characters with > 20% missing data and then removed specimens with > 22% missing data from the remaining set of characters. We then imputed the remaining missing values in the craniodental dataset using the R package *MICE* v.3.16.0 (Van Buuren and Groothuis‐Oudshoorn 2011), employing predictive mean matching with 50 iterations. Imputations were carried out separately for each clade. One skull (AM M.35822) did not match the morphology of skulls from any identified genetic clades and was excluded from the analyses. We also excluded skulls from the southwest of Western Australia, as there was no genetic material available from this region and insufficient skeletal material for meaningful comparisons with the other regions. The final dataset consisted of 28 measurements from 54 skulls.

To minimize the effect of outliers on the PCA, we applied a Shapiro–Wilk Test (Shapiro and Wilk 1965) and log‐transformed variables that were non‐normally distributed and highly skewed (|skew| > 1). Principal component analyses are sensitive to variable scale such that larger measures (e.g., basicranial length) contribute more to the first few principal components than smaller measures (e.g., M2 width) due to their higher variance. Therefore, to control for variation in scale, we conducted a PCA on the correlation matrix to ensure all characters were standardized to the same unit variance (Joliffe 2002). We performed PCA using the pca.calc() in the R package MorphoTools2 (v1.0.1.1) (Šlenker et al. 2022).

In multi‐variate craniodental datasets, overall skull size often dominates the variance explained by PC1 (Umbrello et al. 2023). Size‐independent shape variation has also been successfully used to distinguish morphologically similar species of dasyurids (Viacava et al. 2023). Therefore, to assess shape variation independent of size, we generated a size‐corrected dataset by dividing each measurement by the geometric mean of all measurements (as a proxy for skull size) for that individual, followed by a log‐transformation (Mosimann 1970; Onley et al. 2022; Umbrello et al. 2023). We then performed a PCA on this size‐corrected dataset. To identify variables that best distinguished the three genetic clades identified by this study, a stepwise discriminant analysis (SDA) was conducted using *stepdisc.calc()* in MorphoTools2.

External measurements were taken as follows (Figure 2): body length, anterior tip of nose to cloacal opening; tail length, cloacal opening to posterior tip of hairs on tail; forearm length, posterior of elbow to dorsal surface of wrist when naturally curled over; hindfoot length, posterior of heel to tip of longest toe, ignoring claws; ear length, lower ear notch to top of ear tip; head width, measured at widest point either side of the eyes; head length, anterior tip of nose to posterior of head (occipital region above cervical vertebrae). A Shapiro–Wilk test revealed non‐normal distributions for hindfoot length (eastern: *W* = 0.87, *p* = 0.005; central: *W* = 0.897, *p* = 0.038), brush length (western: *W* = 0.95, *p* = 0.012), and body length (western: *W* = 0.96, *p* = 0.015). We therefore assessed differences between clades using Kruskal–Wallis tests (Kruskal and Wallis 1952) followed by post hoc pairwise Wilcoxon signed‐rank tests (Wilcoxon 1945). To correct for multiple comparisons, we applied a Benjamini–Hochberg correction (Benjamini and Hochberg 1995).

Two names have previously been used to refer to species of kultarr: *Antechinomys laniger* (Gould 1856) and *A. spenceri* Thomas 1906. The holotypes of both are held at the Natural History Museum (NHM), London, UK, and could not be examined due to logistical constraints. To overcome this, we extracted measurements listed in the original descriptions of each species (Gould 1856; Thomas 1906) and compared these to the images of each holotype available on the NHM website (https://data.nhm.ac.uk/dataset/collection‐specimens) to ensure that the two were consistent. To assign species names to the clades identified in this study, we conducted a PCA comparing multivariate trait variation observed in this study to measurements extracted from the original descriptions of the two species. We also compared observed individual measurement ranges with those reported in the original species descriptions.

## 3 Results

### 3.1 Mitochondrial 12S Data

**FIGURE 3:**
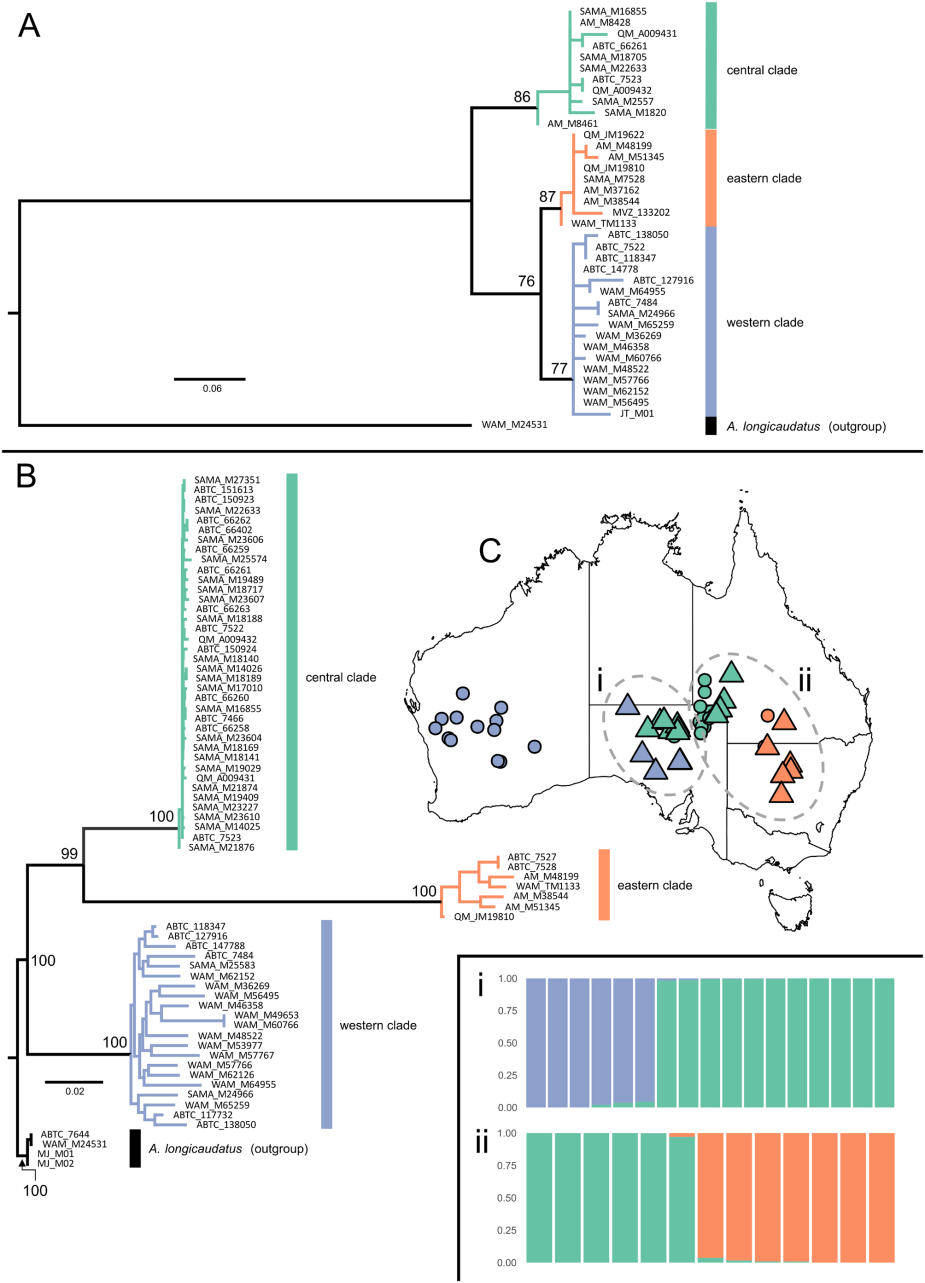
Genetic evidence of three distinct clades of kultarr. (A) Maximum likelihood (ML) phylogenetic tree of mitochondrial 12S sequences (*n* = 38). (B) ML phylogenetic tree of 5036 concatenated SNPs (*n* = 70). Within‐clade ultra‐fast bootstrap values for both analyses not shown. *Antechinomys longicaudatus* (long‐tailed dunnart) samples were used as an outgroup for both analyses. Scale bars show nucleotide differences per site for each tree. (C) Map showing locations of samples, circles are samples included in only the phylogenetic analyses, triangles are samples from the potential admixture zones between the east/central clades, and central/west clades that were included in both the phylogenetic (B) and population admixture (i, ii) analyses.

The mitochondrial alignment included data from 37 kultarr individuals. Of these, 31 were complete sequences from modern specimens (missing data < 5%) and six were partial sequences from historic museum skins and low quality modern genetic material (missing data > 40%). The ML tree based on all mitochondrial sequences identified three kultarr clades. The first consisted of all individuals from central New South Wales and south‐central Queensland (Figure 3). The second clade consisted of all but one individual from the Kati Thanda‐Lake Eyre Basin and Channel Country. The third clade comprised individuals from the Great Victoria Desert and western deserts, along with one individual from Betoota in south‐west Queensland (Figure 3). All three clades were resolved with moderate support (UF Bootstrap support = 87, 86 and 77 respectively).

### 3.2 SNP Data

The SNP‐based phylogeny also identified three clades of kultarr, but with strong support (UFBoot = 100) for all clades (Figure 3). These clades corresponded geographically with those identified in the 12S dataset. Among samples with both SNP and 12S data, clade assignments were congruent in all cases except for sample ABTC7522 from Betoota in southwestern Queensland. This sample clustered within the western clade in the 12S tree but fell within the central clade in the SNP tree (Figure 3). We resequenced 12S from both elutions of the extraction for this sample, and all sequences were identical and matched the western clade.

### 3.3 Population Admixture

Ancestral population reconstruction using sNMF on the population genetics dataset (Table 1) identified *K* = 3 as the most likely scenario, corresponding to the three clades identified in the phylogenetic analyses (Figure 3). Admixture analyses on the two contact zones—between the east/central clades and central/west clades—identified minimal gene flow, with all samples showing less than 5% admixture (Figure 3).

### 3.4 Morphological Data

**FIGURE 4:**
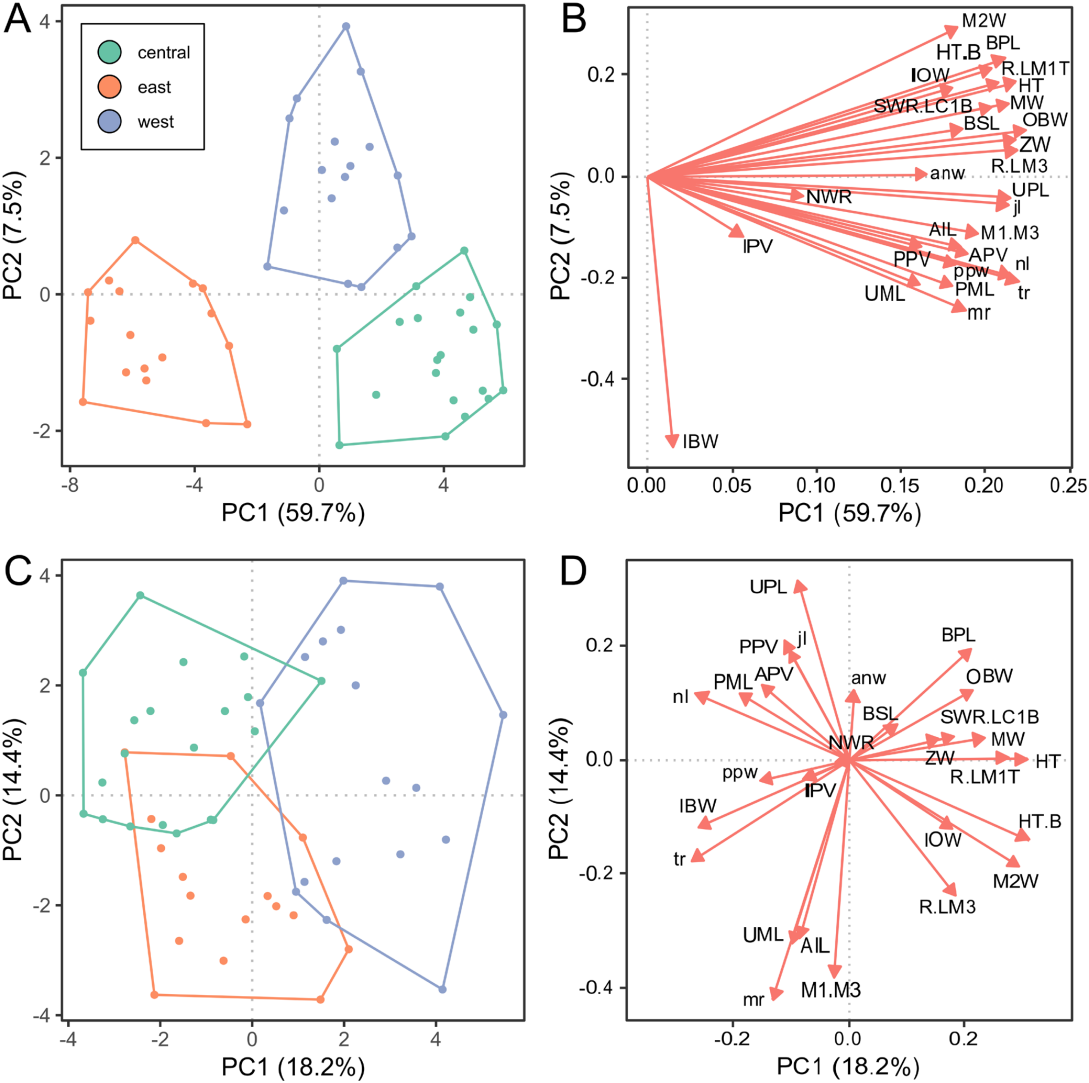
PCA on kultarr craniodental morphology. (A) Craniodental shape variation between the three clades of kultarr on raw data. (B) biplot displaying character eigenvectors which quantify the contribution of each variable to PC1 and PC2 on the raw data; (C) Craniodental shape in kultarr using size‐corrected data to assess shape variation (see text); (D) biplot displaying character eigenvectors which quantify the contribution of each variable to PC1 and PC2 in the size‐corrected dataset.

**FIGURE 5:**
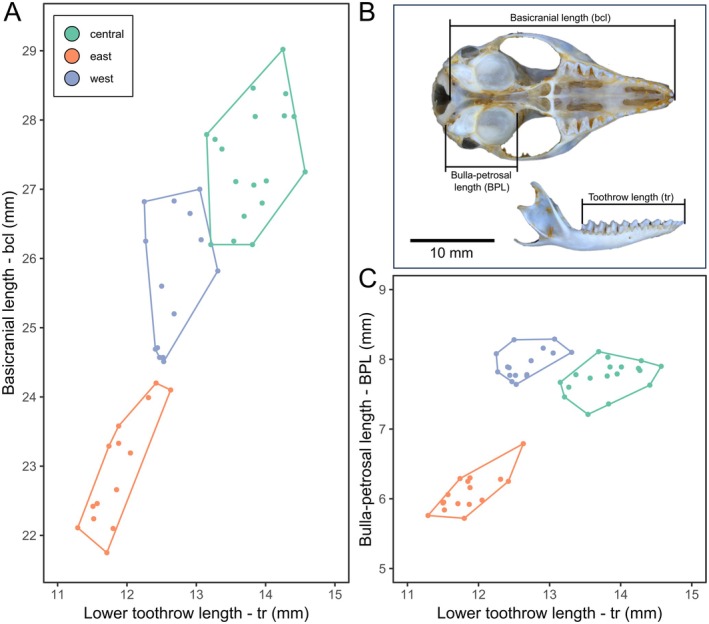
Plots of craniodental measures which best discriminated between the clades of kultarr identified in this study using a stepwise discriminant analysis (SDA). (A) Plot of basicranial length vs. lower toothrow length. (B) Diagram of the three measures which best differentiate the three clades of kultarr identified in this study. (C) Plot of bulla‐petrosal length vs. lower toothrow length.

We examined 270 kultarr specimens (Table A1 in Appendix 1), including 55 skulls (cranium and dentary) and 238 spirit‐preserved specimens. Table A5 in Appendix 1 shows summaries of measurements by clade and sex. The PCA on the imputed craniodental dataset attributed 59.7% of the variance in shape to PC1 and 7.5% to PC2 (Figure 4A). All variables had positive eigenvectors for PC1; hence, PC1 largely represented skull size (Figure 4B). The largest contributor to PC2 was inner bullar width (IBW), indicating that 67.2% of the total variation in craniodental shape could be attributed to differences in overall skull size and auditory bullae size. When skulls were assigned to a clade based on geographic location, there was no overlap between clades in the morphospace represented by PC1 and PC2 in the raw skull shape dataset (Figure 4A). However, when size‐attributable shape variation was removed, the skulls from the three clades overlapped in trait space (Figure 4C), demonstrating the importance of overall size in distinguishing craniodental morphology in this group. In the size‐corrected PCA, PC1 (18.2% variance) was primarily associated with skull width (R.LC1, MW, ZW, SWR.LC1B, R.LM1T) (Figure 4C) and PC2 (14.4% variance) was largely associated with teeth size/toothrow length (UPL, M2W, M1.M3, UML, mr) (Figure 4D). A stepwise discriminant analysis of the raw skull shape dataset identified bulla‐petrosal length (BPL), lower toothrow length (tr), and basicranial length (bcl) as the best characters for distinguishing skulls from the three clades (Figure 5).

**FIGURE 6:**
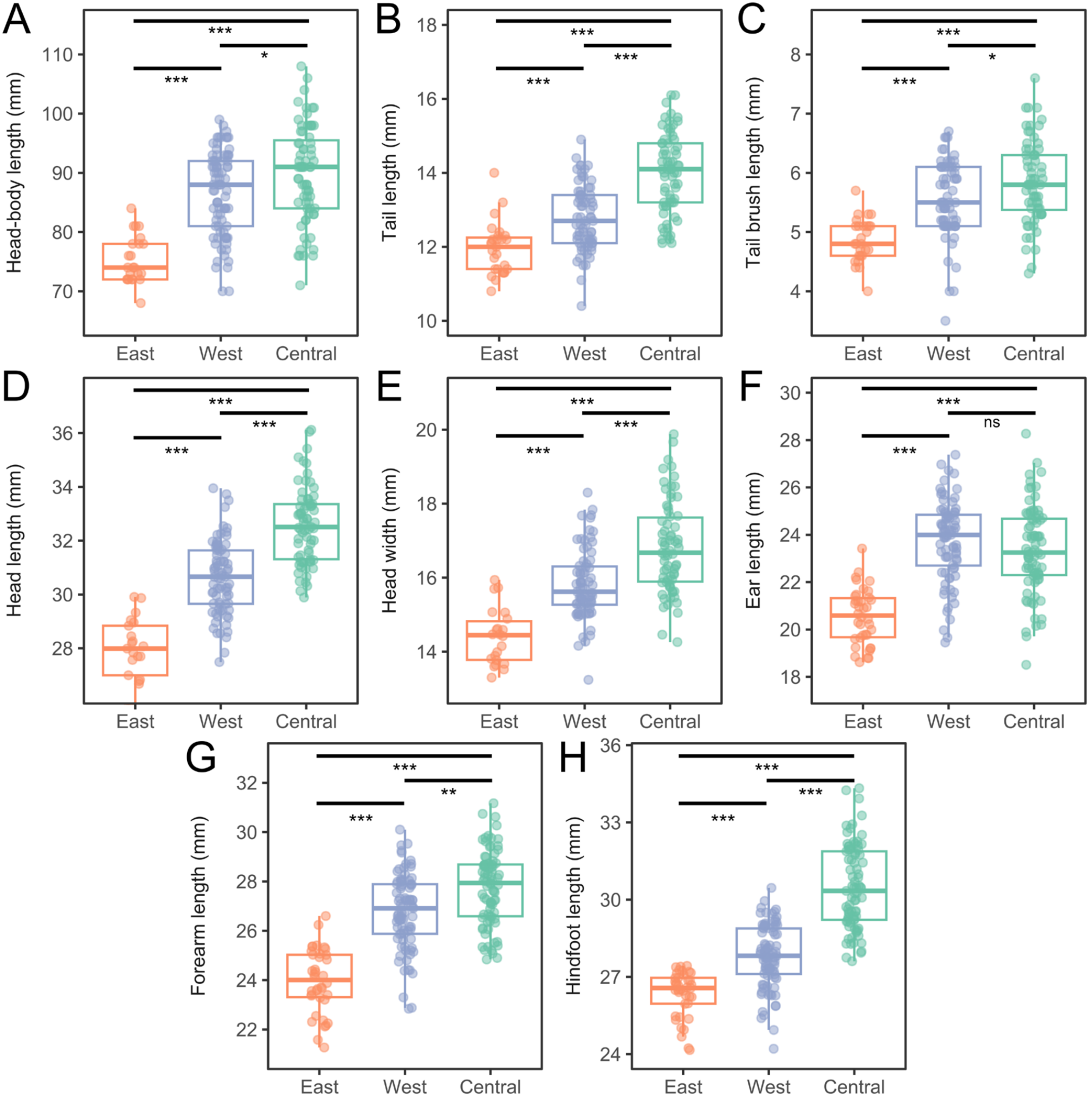
Comparisons of body size measurements across the three clades of kultarr identified in this study. (A–H) Variation in body measurements between clades of kultarr with significant differences between clades shown with asterisks (***, *p* < 0.001; **, *p* < 0.01; *, *p* < 0.05; ns, *p* > 0.05). Significant differences were identified using an in initial Kruskal–Wallis test (Kruskal and Wallis 1952) followed by a post hoc pairwise Wilcoxon signed‐rank tests (Wilcoxon 1945) with a Benjamini–Hochberg correction for multiple hypothesis testing (Benjamini and Hochberg 1995).

Analyses of whole body data found that the eastern clade had significantly smaller ears than the central and western clades (Table A4 in Appendix 1; Figure 6), but no significant difference was detected between the central and western clades (Table A4 in Appendix 1; Figure 6). For all other body measurements, the eastern clade was significantly smaller than the western clade, which was, in turn, significantly smaller than the central clade (Table A4 in Appendix 1; Figure 6). Sexual dimorphism varied across clades. Within the western clade, males were significantly larger than females in body length (*H*(78) = 6.25, *p* = 0.0124), tail length (*H*(69) = 5.4, *p* = 0.0201), hindfoot length (*H*(84) = 13.52, *p* = 0.0002), and forearm length (*H*(82) = 6.54, *p* = 0.0105). Within the central clade, only ear length differed between sexes, with males having significantly larger ears (*H*(73) = 8.72, *p* = 0.0031). Within the eastern clade, males had significantly larger tail length (*H*(25) = 5.14, *p* = 0.0234), hindfoot length (*H*(28) = 4.70, *p* = 0.0302) and forearm length (*H*(28) = 4.70, *p* = 0.0303), though the sample size of females in this clade was small (*n* = 4, vs. *n* = 24 in males).

### 3.5 Taxonomic Conclusions

Based on the data presented here, we conclude that *Antechinomys laniger* consists of at least three independently evolving lineages that constitute different species under the phylogenetic species concept (De Queiroz 2007). This conclusion is supported by several lines of evidence: (1) strong support for monophyly of the eastern, western, and central clades as initially identified by Westerman et al. (2023) using both mitochondrial sequence and nuclear SNP data (Figure 3); (2) no evidence of genetic admixture at the contact zones (Figure 3); (3) distinct multivariate craniodental morphology (Figure 4); and (4) significant differences in external body measures (Figure 6).

### 3.6 Attributing Holotypes

The *A. laniger* holotype was collected from “the interior of New South Wales”, where only the eastern clade occurs (Figure 3). This holotype, as observed from photographs, information in its original description (Gould 1856), and additional information provided in the *A. spenceri* description (Thomas 1906), clearly aligns with the characteristic small skull plus small bullar morphology of the eastern clade as identified by Lidicker and Marlow (1970), Archer (1977), and the present study. Moreover, specimen MVZ133202, which falls within our eastern clade in the 12S phylogenetic tree, was examined by Lidicker and Marlow (1970) and identified as a typical *A. laniger* specimen (Westerman et al. 2023). This specimen is held at the Museum of Vertebrate Zoology, the University of California, Berkeley, USA and therefore was unavailable for examination in this study. Based on genetic, geographic, and morphological evidence, we confidently assign *Antechinomys laniger* sensu stricto to the eastern clade identified by Westerman et al. (2023) and the present study.

**FIGURE 7:**
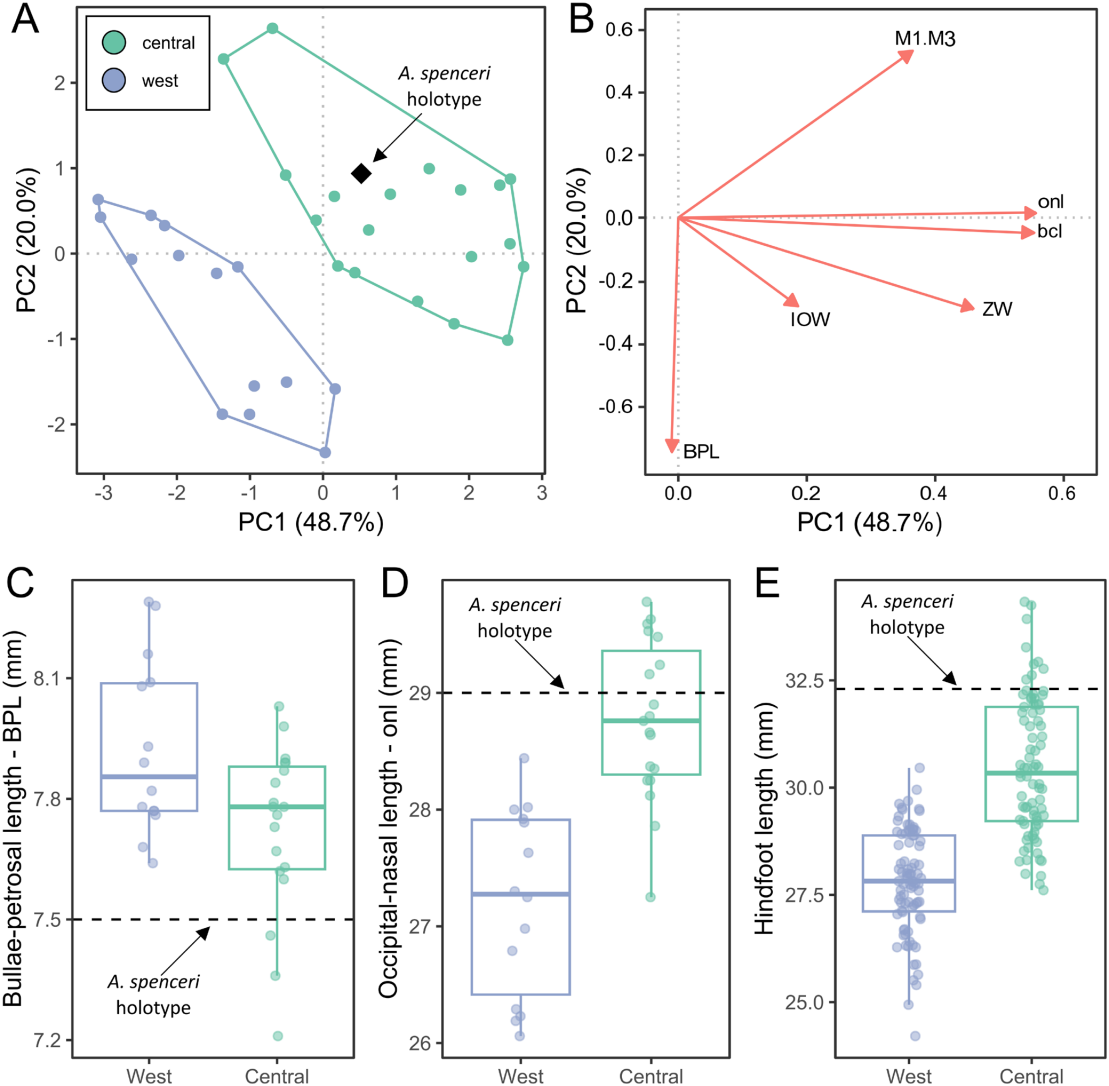
Measurements of the *Antechinomys spenceri* holotype compared to the morphological variation identified in the west and central clades of kultarr identified by this study. (A) PCA of craniodental shape variation in west and central kultarr clades. Coloured circles represent each clade, black diamond represents the *A. spenceri* holotype. (B) biplot displaying character eigenvectors which quantify the contribution of each variable to PC1 and PC2 in the PCA in (A). (C–E) Observed variation in the west and central clades of kultarr identified in this study for: (C) bulla‐petrosal length, (D) occipital‐nasal length, (E) hindfoot length. Dotted lines on (C–E) show the measurements values for the *A. spenceri* holotype. Holotype measurements taken from Thomas (1906).

The *A. spenceri* holotype was collected at Charlotte Waters, Northern Territory, where specimens matching both the western clade and central clade morphologies have been collected. The *A. spenceri* holotype falls within the observed morphospace of the central clade rather than the western clade (Figure 7). Additionally, for occipital‐nasal length (onl), bulla‐petrosal length (BPL), and hindfoot length, the *A. spenceri* holotype falls within the range of values observed for only the central clade (Figure 7). As a result, we confidently assign *A. spenceri* to the central clade. The western clade does not align with any previously described species and is therefore described below as *A. auritus* sp. nov.

## 4 Systematics

**Order Dasyuromorphia** Gill 1872

**Family Dasyuridae** Goldfuss 1820

**Genus *Antechinomys*** Krefft 1867

**Type species: *Phascogale lanigera*** Gould 1856 by subsequent monotypy

**Material examined:** Full list of specimens examined shown in Table A1 in Appendix 1.

**Revised diagnosis:** *Antechinomys* are small sminthopsin dasyurids that possess premolariform canines, inflated alisphenoid tympanic processes (tympanic bullae), inflated petrosals, elongated incisive foramina, elongated maxillopalatine fenestrae, and a tail > 1.25× head‐body length (Archer 1977; Beck et al. 2022; Westerman et al. 2023).

***Antechinomys laniger*** (Gould 1856)

**Synonym: *Phascogale lanigera*** Gould 1856

urn:lsid:zoobank.org:act:3F721EA‐BC5E‐4CEA‐A5D1‐D37E0D32C815

**Recommended common name**: eastern kultarr.

**FIGURE 8:**
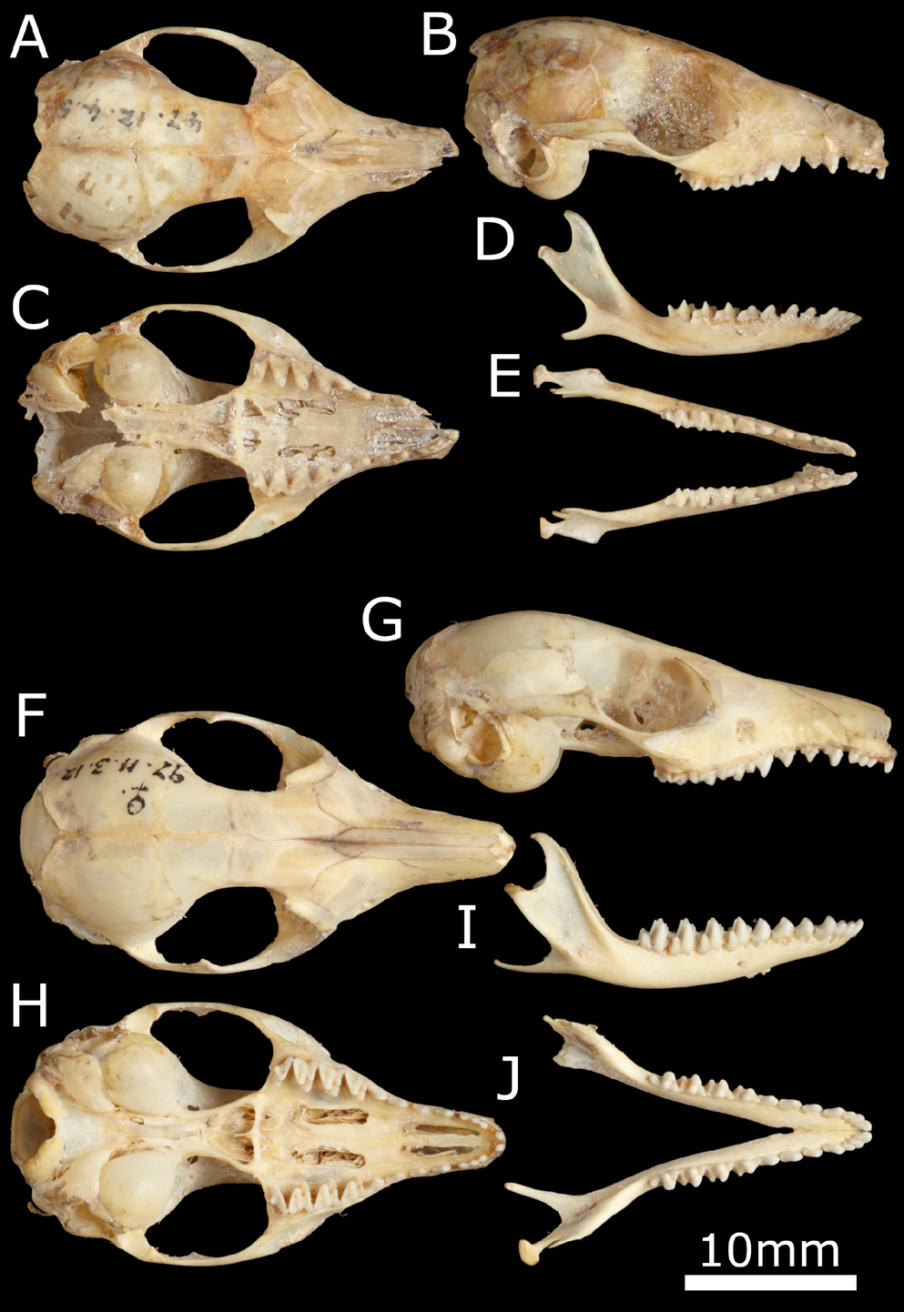
Holotypes of *Antechinomys laniger* (NHM 1847.12.4.5) (A–E) and *A. spenceri* (NHM 1897.11.3.12) (F–J). (A/F) Cranium in dorsal view. (B/G) Cranium in lateral view. (C/H) Cranium in ventral view. (D/I) Mandible in lateral view. (E/J) Mandible in dorsal view. Images available from: https://data.nhm.ac.uk/.

**Holotype:** NHMUK 1847.8.14.22 (skin), NHMUK 1847.12.4.5 (skull). Adult male; Interior of New South Wales, Australia. Collected by T.L. Mitchell (c. 1847). Skull and skin. Specimen held at the Natural History Museum, London UK (Figure 8A‐E).

**Material examined:** Full list of specimens examined shown in Table A1 in Appendix 1.

**Revised diagnosis:** *Antechinomys laniger* sensu stricto is the smallest of the *Antechinomys* species, with all observed specimens having a basicranial length < 25 mm and hindfoot length < 28 mm. *Antechinomys laniger* sensu stricto is diagnosable by its size, the presence of an enlarged granule on the thenar footpad of the manus, and a lack of hair on the ventral surface of the hindfoot. Within the mitochondrial 12S gene, *A. laniger* sensu stricto can be reliably diagnosed from other kultarr species by apomorphic states at sites 89 and 721 (Table A6 in Appendix 1).

**Comparisons to other species:** *Antechinomys laniger* sensu stricto is not sympatric with *A. longicaudatus* ; it may have formally been sympatric with *A. auritus* sp. nov. in the Northern Territory (last record in 1968) (Figure 11); and it is likely sympatric with *A. spenceri* in south‐central Queensland. *Antechinomys laniger* sensu stricto can be distinguished from *A. longicaudatus* by the absence of the hallux on the hindfoot, complete fusion of the interdigital pads on the pes, elongated limbs, elongated tail with a terminal black brush, inflated lacrimals, and wider molars. It can be distinguished from *A. auritus* sp. nov. and *A. spenceri* by its smaller size, shorter ears, presence of four pairs of nipples in females, less inflated alisphenoid tympanic processes and petrosals, and less elongated snout.

**Distribution and habitat:** *Antechinomys laniger* is found in the open woodlands of central New South Wales and south‐central Queensland, with almost all modern records falling within the Mulga Lands or Cober Peneplain IBRA Regions (DCCEEW 2020). Historical records and specimens from Wilcannia, Menindee, Mildura, and Deniliquin, Walbundrie, Walgett (all NSW), and Boyne River and Yulabilla (both Qld) suggest a previous distribution spanning most of semi‐arid eastern Australia (Figure 10). Historical specimens from near Tobermorey Station, Roper River, and near Tennant Creek (all NT) and Cloncurry (Qld) match the morphology of the eastern *A. laniger* specimens, suggesting that the distribution of this species once continued northwards, potentially around the northern edge of the Kati Thanda‐Lake Eyre Basin (Figure 10). The records from Tennant Creek, NT are from the Spencer–Gillen NT Expedition in 1901, but it is unclear exactly where in the vicinity of Tennant Creek the specimens were collected. We have provisionally assigned these Northern Territory specimens to *A. laniger* based on the morphological data as no genetic data is available to confirm the species identity due to the age and condition of these specimens.

***Antechinomys spenceri*** Thomas 1906 reinst. stat.

urn:lsid:zoobank.org:act:1ADE904C‐381A‐4771‐A306‐F4CB67EE54F2

**Recommended common name**: gibber kultarr.

**Holotype:** NHMUK 1897.11.3.12. Adult female; Charlotte Waters, central Australia. Collected by B. Spencer (c. 1897). Skull only. Specimen held at the Natural History Museum, London UK. Figure 8F‐J.

**Material examined:** Full list of specimens examined shown in Table A1 in Appendix 1.

**Revised diagnosis:** *Antechinomys spenceri* is the largest of the *Antechinomys* species, with all observed adult specimens having a lower toothrow length > 13 mm, basicranial length > 26 mm, and hindfoot length (long pes) > 27 mm and often > 30 mm. *Antechinomys spenceri* is generally distinguishable from other *Antechinomys* by its larger size, elongated hindlimbs, and more robust (thicker) head and limbs. However, there is substantial morphological overlap with *A. auritus* sp. nov., and only dental morphology (presence of blade running lingually from tip of the StD on the M1 in *A. auritus* sp. nov.) and genetic data can currently distinguish them with confidence. Within the mitochondrial 12S gene, *A. spenceri* can be reliably diagnosed from other kultarr species by apomorphic states at sites 89, 161, 165, 177, 212, 263, 384, 447, 493, 942, and 947 (Table A6 in Appendix 1).

**Comparisons to other species:** *Antechinomys spenceri* is not sympatric with *A. longicaudatus* , due to the latter's preference for mountainous rocky outcrops; it is likely sympatric with *A. laniger* in south‐central Queensland; and it is sympatric with *A. auritus* sp. nov.in the western Simpson Desert, Finke River (Larapinta) Basin and southern Macdonnell Ranges. It can be distinguished from *A. longicaudatus* by the absence of the hallux on the hindfoot, complete fusion of the interdigital pads on the pes, elongated limbs, elongated tail with a terminal black brush, inflated lacrimals and wider molars. It can be distinguished from *A. laniger* by its larger size, longer ears, presence of three pairs of nipples in females, more inflated alisphenoid tympanic processes and petrosals and more elongated snout. It can be distinguished from *A. auritus* sp. nov. by its larger size, more robust (thicker) limbs and head, elongated hindfeet, and the absence of a blade running lingually from tip of the StD on the M1.

**Distribution and habitat:** *Antechinomys spenceri* is found in the extensive gibber (stony) deserts of central Australia. The distribution of *A. spenceri* roughly corresponds to the Georgina and Diamantina River basins in southeast Queensland, the Sturt Stony Desert in northeast South Australia and the extensive gibber plains running north‐westwards from Kati Thanda‐Lake Eyre to the MacDonnell Ranges around Alice Springs. This distribution falls mostly within the Channel Country and Stony Plains IBRA Bioregions (DCCEW 2020), with some likely records in the Finke Region. The northeastern‐most confirmed records of *A. spenceri* come from Diamantina National Park. The northwestern‐most records are listed as from ‘Alice Springs’; however, no more specific location data are available. The extent of *A. spenceri* occurrence in the western Simpson Desert, Finke River basin and MacDonnell Ranges in the southern NT is unclear and will require further work in the field. Kultarr in central Australia have almost exclusively been caught on gibber plains in surveys in both the western Simpson Desert (Pavey et al. 2020) and Sturt Stony Desert (Brandle 1998). Many of these records cannot be assigned to either *A. spenceri* or *A. auritus* sp. nov., and there is a conspicuous lack of records from the nearby Simpson‐Strzelecki Dunefields, emphasizing the importance of gibber plains as the primary habitat for *A. spenceri* .

***Antechinomys auritus* sp. nov.**

urn:lsid:zoobank.org:act:98C72CC9‐B0D8‐4762‐803E‐50D31569ADBA

**Recommended common name**: long‐eared kultarr.

**FIGURE 9:**
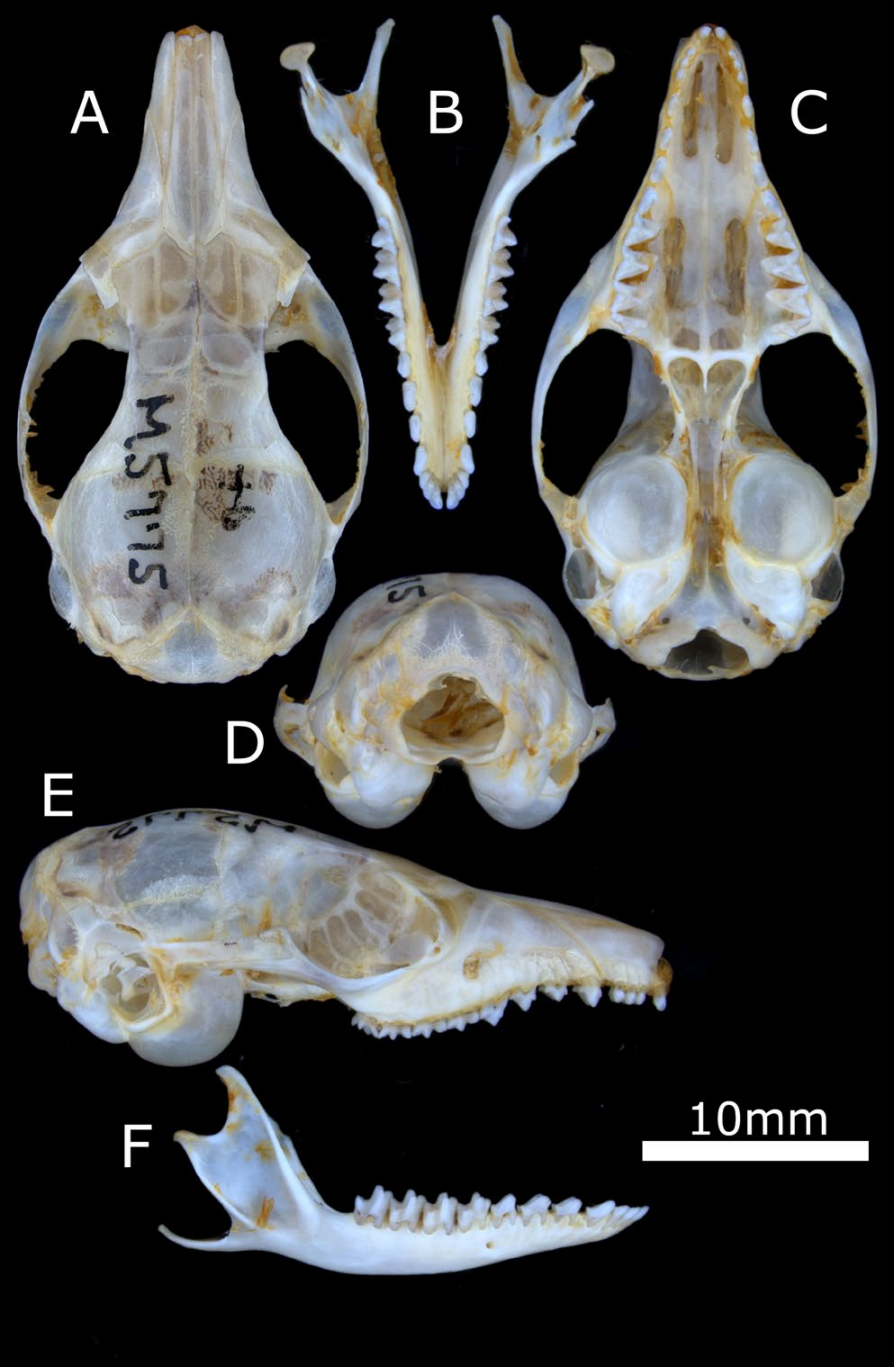
Skull of the holotype of *Antechinomys auritus* sp. nov. (WAM M5775). (A) Cranium in dorsal view. (B) Mandible in dorsal view. (C) Cranium in ventral view. (D) Cranium in posterior view. (E) Cranium in lateral view. (F) Mandible in lateral view.

**Holotype:** WAM M5775, an adult female from Elder Creek, Western Australia (−26.1166, 126.5833). Collected by “Aboriginal children” [*sic*] and given to M. de Graaf (c. Jul‐Aug 1963). Spirit and skull. Specimen held at the Western Australian Museum, Western Australia. Figure 9.

**Paratypes:** WAM M5776: Adult male; Elder Creek, Western Australia (−26.1166, 126.5833). Collected by “Aboriginal children” [*sic*] and given to M. de Graaf c. Jul–Aug 1963. Spirit and skull. WAM M56495: Adult female; Lake Mason, Western Australia (−27.6975, 119.28). Collected by M. Cowan and R. How, 20 Sep 2005. Spirit only. Liver tissue. WAM M57766: Adult male; Weld Range, Western Australia (−26.9833, 117.4667). Collected by S. Ford and V. Cartledge, 18 Oct 2006. Spirit only. Liver tissue. WAM M23674: Adult male; Mileura Homestead, Western Australia (−26.26, 117.33). Collected by T. Valente, 1986. Spirit and skull. SAMA M24886: Adult female; Tarcoola, South Australia (−29.8847, 133.5669). Collected by M. Clunies‐Ross, 7 Nov 2011. Spirit only. SAMA M26298: Adult male; Kingoonya, South Australia (−31.2197, 134.7839). Collected by C. Lynch, 19 May 2012. Spirit only. SAMA M24966: Juvenile female; Donald's Well, South Australia (−26.1864, 132.3478). Collected by M. Ward, 22 Mar 2011. Spirit and skull. Liver tissue: ABTC139261.

**Etymology:** The species name *auritus* means ‘eared’ or ‘long‐eared’ in Latin. The common name similarly refers to its remarkably large ears relative to its body size compared to other kultarr species.

**Material examined:** The full list of specimens examined is listed in Table A1 in Appendix 1.

**Revised diagnosis:** *Antechinomys auritus* has the largest ears of any *Antechinomys* species relative to head size and the most inflated alisphenoid tympanic processes (auditory bullae) and petrosals of any sminthopsin. It also possesses a blade running lingually from the tip of the StD on the M1 not found in other *Antechinomys* species and can be externally diagnosed by the combination of large ears, an elongated snout, and thin limbs—*Antechinomys spenceri* has notably more robust (thicker) limbs and feet. Diagnosis from juvenile or subadult *A. spenceri* specimens may be challenging, and only genetic data can currently distinguish them with confidence outside of tooth morphology. Within the mitochondrial 12S gene, *A. auritus* can be reliably diagnosed from other kultarr species by apomorphic states at sites 89, 392, and 492 (Table A6 in Appendix 1).

**Comparisons to other species:** *Antechinomys auritus* is not sympatric with *A. longicaudatus* , due to the latter's preference for mountainous rocky outcrops; it may have formally been sympatric with *A. laniger* sensu stricto in the Northern Territory (last record in 1968) (Figure 11); and it is sympatric with *A. spenceri* in the western Simpson Desert, Finke River (Larapinta) Basin, and southern Macdonnell Ranges. It can be distinguished from *A. longicaudatus* by the absence of the hallux on the hindfoot, complete fusion of the interdigital pads on the pes, elongated limbs, elongated tail with a terminal black brush, inflated lacrimals, and wider molars. It can be distinguished from *A. laniger* by its larger size, longer ears, presence of three pairs of nipples in females, more inflated alisphenoid tympanic processes and petrosals, and more elongated snout. It can be distinguished from *A. spenceri* by its smaller size, less robust (thinner) limbs and head, shortened hindfeet, and the presence of a blade running lingually from the tip of the StD on the M1.

## 5 Description

### 5.1 Pelage

The fur on the back is up to 11 mm long, with the basal 7–8 mm almost black, median 2–3 mm a light brown, and the distal 1 mm a dark brown. This gives the back an overall light brown appearance, with flecks of darker brown coming through it depending on the hair orientation. The fur becomes slightly lighter on the flanks before becoming almost pure white on the belly. There is a darker patch on the back of the head that continues forward between the ears to just level with the eyes, where a dark eye ring is present. The basal approximately two‐thirds of the tail is covered in short (< 1 mm) hairs, which culminate in a pencil of dark brown hairs up to 20 mm long at the caudal tip of the tail. A dark brown stripe runs along the ventral surface of the tail and hindfeet. *A. spenceri* tends to be more light brown in colour, with a less pronounced dark facial marking that does not reach between the eyes as it does in *A. auritus* . *Antechinomys laniger* tends to be darker in colour than *A. auritus* . While there appears to be a tendency for *A. spenceri* to be lighter in colour and *A. laniger* to be darker, there is also geographic variation in pelage colour among all three species that has not been properly documented, and further work is needed to confirm the diagnostic qualities of pelage colour between these species.

### 5.2 Vibrissae

Approximately 18 mystaceal vibrissae are present on either side of the head which are approximately 32 mm long. Around five supra‐orbital vibrissae are present, and these are slightly shorter than the mystaceal vibrissae (~27 mm). As with all species of kultarr, 3–4 very long (> 30 mm) vibrissae derive from the lateral side of the ankle. The function of these is not well understood, but they are assumed to be analogous in function to the mystaceal vibrissae. In *A. longicaudatus* , the facial vibrissae are longer than in kultarr species, but the ankle vibrissae are much shorter.

### 5.3 Tail

The tail is slightly longer than the head and body and shows no sign of thickening at the tail base. The caudal ~40% of the tail is covered with a dark brush. Relative to body size, tail length is similar in all species of kultarr, and there have been no observed differences in brush morphology between species. *Antechinomys longicaudatus* has a tail much longer than its body length (> 2× body length) and lacks the caudal brush of all kultarr species, instead just having a small tuft of hair at the very tip of the tail.

### 5.4 Hindfoot (pes)

The hindfoot is elongated and the hallux is absent as in all species of kultarr. The interdigital pads are fused into a singular tri‐lobed pad. The ventral surface of the interdigital pads is granular, with granules slightly elongated, giving a rounded pyramid shape. The interdigital pad is mostly hairless, with only the ventral base of the pad haired. The rest of the ventral surface of the hindfoot is covered in silvery hairs, with the ventral surface of the toes and either side of the interdigital pad being particularly hairy. The pes morphology is largely similar between species of kultarr, with the main differences being *A. laniger* having a much less hairy footpad than *A. auritus* and *A. spenceri* , and *A. spenceri* having elongated hindfeet relative to the other species of kultarr. *Antechinomys longicaudatus* has a similarly trilobed footpad, but each lobe has a large, elongated granule running towards the heel with many clear transverse striations on each. The hallux is also present on *A. longicaudatus* as well as an enlarged large posthallucal pad.

### 5.5 Forefoot (manus)

The interdigital pads of the manus are partially fused and covered with large granules which join the pads together. The granules continue to the toe pads. The ventral surface of the manus surrounding the granules is haired but not to the same extent as the pes. *Antechinomys spenceri* has a largely similar manus morphology to *A. auritus* ; however, *A. laniger* possesses an enlarged thenar granule on the hindpad. The manus of *A. longicaudatus* has enlarged striated granules on each footpad, similar to the pes in this species.

### 5.6 Ear

The ear is very large, and the pinnae are fringed with pale hairs. The supratragus is slightly curled. The ear is much smaller in *A. laniger* and *A. longicaudatus* .

### 5.7 Nipple number

All known adult female *A. auritus* specimens have three pairs of nipples, as with *A. spenceri* and *A. longicaudatus* , differentiating these two species from *A. laniger* , which has four pairs. Archer (1977) reports specimens of *A. laniger* with five pairs of nipples and specimens of *A. auritus* /*spenceri* with two pairs; however, both appear to be abnormal.

### 5.8 Cranium

The holotype skull is in good condition other than some slight tooth wear and small amounts of tissue remaining around the teeth and some foramina.

In dorsal view, the nasals protrude slightly posteriorly past the anterior tip of the frontals, such that the nasal‐frontal suture is positioned slightly posterior to the anterior rim of the orbit. In *A. laniger* , the nasals protrude even further posteriorly but not so in *A. spenceri* and *A. longicaudatus* , with both having a nasal‐frontal suture that is approximately level with the anterior rim of the orbit. The frontal–parietal suture is mostly straight meso‐laterally. The anterodorsal part of each frontal is convex, creating an anteroposterior depression leading from the anterior tip of the nasals to the anterior of the parietal. The interparietal is not visible in any species of *Antechinomys*, and the parietal‐supraoccipital suture forms an anterior facing triangle with the antero‐dorsal part of the supraoccipital sitting between the two parietals. The lacrimals of all kultarr species are enlarged and possess a strong ridge along the dorsal edge of the orbital rim, whereas the lacrimals of *A. longicaudatus* are always small and unridged.

In lateral view, the snout is elongated by an enlargement of the premaxilla. *Antechinomys spenceri* shows similar elongation of the premaxilla, but the premaxilla is reduced in *A. laniger* and *A. longicaudatus* . *Antechinomys longicaudatus* instead exhibits elongation of the maxilla, giving a similar overall snout length to *A. auritus* and *A. spenceri* . The lateral face of the maxilla is mostly flat in all kultarr species, but it is curved posterolaterally in *A. longicaudatus* . The infraorbital foramen contains one anterior foramen on the dorsal surface of the maxilla; *A. laniger* may possess multiple anterior foramina here. In *A. longicaudatus* , the infraorbital foramen is more tubular, with a ridge running posteromedially from its dorsal edge. Two lacrimal foramina are present, positioned on the lateral and posterolateral surfaces respectively. In *A. laniger* , the lateral lacrimal foramen is enlarged, and the posterolateral foramen is highly reduced or absent. In *A. longicaudatus* , both foramina are positioned on the lateral surface of the lacrimal.

The zygomatic arch is relatively thin, whereas *A. laniger* shows a pronounced thickening on the anterior part of the squamosal, giving a more robust zygomatic arch in this species. The sphenorbital fissure is separated from the foramen rotundum by a wide bar of the alisphenoid. This bar is much thinner in *A. laniger* and *A. longicaudatus* . The ventral surface of the bullae protrudes substantially ventral to the molar row but is level with, or only just ventral to, the molar row in *A. laniger* and *A. longicaudatus* . The petrosals are clearly visible laterally but only just so in *A. laniger* . The bullae and petrosals are highly inflated, as is seen in *A. spenceri* ; however, only moderate inflation is seen in *A. longicaudatus* , with little to none in *A. laniger* . All species of *Antechinomys* display frontal‐squamosal contact.

In ventral view, the incisive foramen ends posterior to the anterior tip of the P2. *Antechinomys spenceri* has a larger premolar diastema, leading to the incisive foramina being longer overall, whereas *A. longicaudatus* and *A. laniger* have smaller premolar diastemata. The maxillo‐palatine fenestrae begin level with the posterior tip of the P3; however, in A. laniger, they start level with the anterior tip of the P3. The maxillo‐palatine fenestrae finish at the midpoint of the M3 in all species and are wider in *A. spenceri* . Maxillary foramina have been observed in some specimens (e.g., WAM M23674, WAM M5776) as well as one specimen of *A. laniger* (NMV C6946). The palatal foramen is large and is reduced in *A. laniger* . The anterior end of the presphenoid is variably expanded meso‐laterally. The carotid foramen opens within the basisphenoid; however, it is positioned between the basisphenoid and alisphenoid in *A. spenceri* . The transverse foramen is smaller in *A. auritus* and *A. spenceri* than it is in either *A. laniger* or *A. longicaudatus* . A secondary foramen ovale may be present within the alisphenoid within *A. auritus* only; in these cases, the primary foramen ovale is absent. When present, the primary foramen ovale is highly narrowed mesolaterally and is often completely obscured by the alisphenoid in the ventral view. The primary foramen ovale is always present in *A. spenceri* and *A. longicaudatus* but remains highly reduced in both, whereas it is much larger in *A. laniger* , allowing the interior of the braincase to be clearly visible ventrally. The bullae and petrosals are considerably inflated, with the bullae being particularly inflated anteriorly. In *A. spenceri* , the bullae are also inflated; however, they are less rounded anteriorly. The petrosals are also wider posteromedially in *A. spenceri* . The petrosals and bullae are small in *A. laniger* and intermediate in size in *A. longicaudatus* . The paroccipital process is large but variable in shape and tends to be more pointed posteriorly in *A. laniger* and *A. longicaudatus* . The jugular foramen and the foramen for the inferior petrosal sinus are similar in size, with the former being more rounded. Two hypoglossal foramina are present and equal in size, but the anterior foramen is much larger than the posterior foramen in *A. laniger* .

In posterior view, the foramen magnum is similar in size across all species of *Antechinomys*, tending to be slightly smaller in *A. spenceri* and slightly larger in *A. laniger* .

### 5.9 Dentary

Small diastemata are present between the premolars of *A. auritus* ; these diastemata are larger in *A. spenceri* and smaller in *A. laniger* . The mental foramen is variably positioned from beneath the p2 to beneath the m1; however, it is consistently beneath the m1 in *A. spenceri* and *A. longicaudatus* . The anterior edge of the ascending arm of the ramus begins wide, thinning dorsally; however, it maintains a consistent thickness in *A. longicaudatus* . The angle of the ramus is more obtuse in *A. auritus* and *A. spenceri* than in *A. laniger* and *A. longicaudatus* . The angular process of the dentary is thicker in *A. spenceri* relative to the other species of *Antechinomys*. The mandibular foramen is larger in *A. auritus* and *A. spenceri* relative to *A. laniger* and *A. longicaudatus* .

## 6 Dentition

### 6.1 Upper Incisors

The I1 is slightly procumbent with no anterior curvature, as in all *Antechinomys* except *A. longicaudatus* , which has a pronounced anterior curvature and has two blades running down the posterior edge either side of a central groove. It is twice as tall as the I2–4 and subequal in crown height to the C1. The I1–4 are subequal in length, as in other *Antechinomys* except *A. laniger* , where they increase in length posteriorly. The I1–4 are similar in absolute size to *A. laniger* but smaller than in *A. longicaudatus* and *A. spenceri* . The I2–3 are roughly pentagonal, with a central cusp bladed to the anterior and posterior tip before pinching inwards to the root. I4 is similar in shape but is bulged slightly anteriorly to meet I3. Posterior buccal cingula are absent from the I2 to I3 but present on the I4, which also has a highly reduced posterior cusp.

### 6.2 Upper Canines

The C1 is separated from the I4 by a diastema and is premolariform with a small anterior cusp and large posterior cusp. The anterior cusp is smaller in *A. auritus* than in *A. spenceri* and *A. laniger* , which are smaller still than in *A. longicaudatus* . The C1 is approximately equal in crown height to the P3 (slightly taller than P3 in *A. spenceri* and *A. longicaudatus* , much taller than P3 in *A. laniger* ). The C1 of *A. longicaudatus* is different in shape from other *Antechinomys*, with the crown being wider and less pointed despite being taller. The C1 has narrow anterior cingula, wide posterobuccal cingula, and no lingual cingula as in all *Antechinomys*.

### 6.3 Upper Premolars

The upper premolars are narrow and widely spaced and increase posteriorly in crown height. There are diastema present between the C1 and each of the premolars. The diastema are longer in *A. spenceri* and smaller in *A. laniger* , with no diastema present between the P2 and the P3 of *A. laniger* . There are anterior buccal and lingual cingula present on the P1‐3; however, they are much reduced on the P1 and the P3. There are posterior buccal and lingual cingula as well as small anterior cusps present on the P1‐3. Large posterior cusps are present on the P1‐3 with the posterior cusp being enlarged but flattened on the P3, making it hard to distinguish from the posterior edge of the enlarged posterior cingulum. The posterior main blade in reduced in P3, giving no distinction between the posterior buccal and lingual cingula.

### 6.4 Upper Molars

In the M1, a stylar crest runs from the anterior tip of the StA lingually along the edge of the small anterior cingulum. The posterior tip of the P3 meets this blade below and lingually to the StA. Stylar cusp B is present but much reduced. There is a blade running posteriorly from the StB that shows no evidence of a StC and meets the base of the very large StD. Stylar cusp E is very small and only observable in unworn specimens. The metacone is immediately lingual to the StD and is slightly shorter than it but much wider buccolingually. There is a short blade (marked with an asterisk in Figure 2F) running lingually from the tip of the StD towards the centrocrista (postparacrista and premetacrista) in *A. auritus* , which is not present in other species of *Antechinomys*. A stylar crest runs from the StD to the metastyle via the StE. The paracone is reduced and slightly taller than the protocone. The preprotocrista runs anterobuccally and ends at the base of the paracone, where there is no protoconule. The postprotocrista descends steeply on the posterior side of the protocone and ends at the base of the metacone, where there is no metaconule. The M1 morphology is largely consistent within *Antechinomys*, although the M1 is slightly narrower in *A. laniger* and slightly wider and longer in *A. spenceri* . The StB is much larger in *A. longicaudatus* , and the StD is much smaller relative to other *Antechinomys*. A parastyle is also present in *A. longicaudatus* .

The M2 is similar to the M1 with the following differences: the anterior cingulum is narrower but longer along the lingual axis; the StA and the stylar crest running posteriorly from it are shifted slightly buccally, leading them to protrude out lingually past the lingual edge of the StD; the posterior edge of this blade is more defined, making the StB present but joined to the StA by the aforementioned blade; the paracone is shifted lingually, leading to a longer preparacrista; the StD is more pointed, more circular, and less flared out at the base and shifted slightly anteriorly in position; the blade running lingually from the StD towards the centrocrista is shorter; the metacone is slightly smaller; the metastyle of the tooth is also slightly elongated relative to M1, and the protocone is taller, leading to a steeper postprotocrista. The blade running lingually from the tip of the StD towards the centrocrista remains absent on the M2 of *A. spenceri* and *A. longicaudatus* but is present in *A. laniger* . In *A. spenceri* , a minute StC is present posterior to the StB, and the tooth is overall slightly longer and wider. In *A. laniger* , the M2 is slightly shorter and much narrower than in *A. auritus* .

The M3 is similar to the M2 with the following differences: the anterior cingulum is further narrowed and elongated lingually; the StA and the stylar crest running posteriorly from it are shifted further buccally, creating a near right angle of the buccal edge of the tooth where the blade meets the base of the StD; the StB is smaller and more rounded; the preparacrista is elongated due to the buccal shifting of the StA and its stylar crest; the StD is reduced, decreasing the angle of the blade running lingually from the tip of StD; the protocone is shorter and the postprotocrista is more curved than in M2; the metastyle is reduced, making the distance from StD to the posterobuccal tip of the tooth shorter than in M2; the stylar crest between the StD and the metastyle is absent, with only a small StE present. In *A. spenceri* , the StE is absent and the blade running lingually from the tip of StD towards the centrocrista is now present. The M3 is also longer and wider in *A. spenceri* and *A. longicaudatus* but shorter and narrower in *A. laniger* , and the curvature present in the postprotocrista of the *A. auritus* M3 is absent in both *A. spenceri* and *A. laniger* . In *A. longicaudatus* , the StD and the StE are reduced, with the StB being the tallest of the stylar cusps.

The M4 is highly reduced overall. The anterior cingulum is further reduced in width from the M3 but is similar in length. The paracone is larger, being of similar width to the metacone of the M3, and the preparacrista is the longest of all molars, with it becoming progressively longer from M1 to M4. The StA and the StB are present, and 0–3 min cusps are variably present in the depression posterior to the StB. The anterior cingulum is wider in *A. spenceri* and narrower in *A. laniger* , with both also lacking any extra cusps posterior to the StB. The M4 is slightly wider in *A. spenceri* , slightly shorter in *A. longicaudatus* , and much smaller in *A. laniger* . In *A. longicaudatus* , the postparacrista is shorter and the posterior cusp is reduced.

### 6.5 Lower Incisors

All lower incisors are positioned horizontally from the tip of the dentary. The i1 is taller crowned than the i2–3, which are equal in crown height. The i1 anterior blade lines up with the antero‐lingual edge of the i2 and the lingual edge of the i3. The i1 and the i2 have a small posterior cusp, which is larger in the i3. In *A. spenceri* , the i1 is wider but shorter; the i2 is wider and of a similar length, and the posterior cusp on the i3 is much larger. In *A. laniger* , i‐3 are narrower and shorter, and the posterior cusp on i3 is smaller. In *A. longicaudatus* , i1–3 are similar in size to *A. spenceri* , but with a much‐reduced posterior cusp on the i3.

### 6.6 Lower Canines

The c1 is angled horizontally so that the crown lies over the top of the root of the i3. The c1 has no anterior cusp and is curved anteriorly with a large posterior cusp. The height of the c1 is greater than the i1 and equal to the p1. There are posterior buccal and lingual cingula present, but the buccal is much wider and more distinct. In *A. spenceri* and *A. longicaudatus* , the c1 is larger and taller, with a proportionately much smaller posterior cusp. In *A. laniger* , the c1 is shorter in crown height as well as crown length and is less anteriorly curved than in *A. auritus* .

### 6.7 Lower Premolars

The p1 and the p3 are narrower than the c1, with the p2 similar to the c1 in width. The p2 and c1‐p3 are evenly spaced with diastema between each tooth. Lower premolar crown height increases posteriorly. The anterior cusps are highly reduced on the p1‐p2 and the primary cusp of both is shifted anteriorly, displaying strong anterior curvature and an overall tooth shape similar to the c1. The anterior cusp is much larger on the p3. Anterior cingula are absent from the p1, but present on the p2 where the anterior lingual cingulum is wider than the anterior buccal cingulum and the two do not meet. The anterior lingual and buccal cingula are connected in the i3. Posterior cusps and cingula are present and similarly sized in all lower premolars, with the p3 posterior cingula crested posteriorly. In *A. spenceri* , all lower premolars are slightly longer than in *A. auritus* . The anterior cusps on the p1 and the p2 are larger in *A. spenceri* , but the p3 anterior cusps are smaller. The ‘c1‐like’ anterior curvature seen in the p1‐p2 of *A. auritus* is present in all premolars in other *Antechinomys*. In *A. laniger* all premolars and the diastema between them are slightly shorter than in *A. auritus* . The anterior cusps of the p1‐p3 in *A. laniger* are all small. In *A. longicaudatus,* the premolars are similar in length to those in *A. auritus;* however, the anterior cusps are greatly reduced. A second tiny cusp directly anterior to the main anterior cusp is present on the premolars of unworn specimens of *A. longicaudatus* .

### 6.8 Lower Molars

All lower molars are relatively wide, increasing from the m1 to m3 in width, with the m4 equal to the m3 in width. In the m1, the talonid is wider than the trigonid, and a narrow anterior cingulum is present. A small lingual cingulum is also present as well as a posterior cingulum. The protoconid and metaconid are close, giving a small and almost absent metacristid. The posthypocristid is long and slightly curved. The cristid obliqua meets the trigonid at the midpoint of the metacristid. The protoconid is large and approximately twice the height of the paraconid. The metaconid is slightly shorter than the protoconid. The paracristid is strongly bladed with a strong carnassial notch. The entoconid is absent. In *A. spenceri* , all lower molars are longer but similar in width, with the paraconid of the m1 enlarged and shifted anteriorly. In *A. laniger* , all lower molars are shorter and narrower than in *A. auritus* , and the m1 has a wider posterior cingulum. The paraconid is also closer to the metaconid on the m1 of *A. laniger* , and the trigonid is shorter.

The m2 is similar to the m1 other than the following: the talonid is only marginally wider than the trigonid; the buccal cingulum is reduced; the hypoconulid is enlarged and flattened on the lingual side, making the distinction between the posterior cingulum and the lingual slope of the hypoconulid less distinct; the paraconid and metaconid are both taller and more so than the protoconid; the paraconid is shifted slightly posterolingually and the protoconid is enlarged lingually, giving a slightly obtuse angle between the paraconid and protoconid about the metaconid; the paracristid blade runs posteriorly from the paraconid rather than anteriorly from the protoconid; the cristid obliqua meets the trigonid slightly more lingually. In *A. spenceri* , the m2 is longer and wider, but the cusp morphology is largely similar. In *A. longicaudatus* , the m2 is similar in size and shape to that of *A. auritus* . In *A. laniger* , the m2 is shorter and narrower than in *A. auritus* , and a small entoconid is present.

The m3 is similar to the m2 other than the following: the talonid and trigonid are equal in width; the protoconid is shifted slightly anterolingually, giving a slightly longer metacristid; the hypoconid is slightly reduced and shifted anteriorly; the posterior cingulum is wider; the hypoconulid is larger; the buccal end of the posthypocristid forms a small ridge running directly posteriorly to meet the hypoconulid, creating a near 90° angle between the hypoconulid and hypoconid about the buccal tip of the posthypocristid.

The m3 is longer and wider in *A. spenceri* , and shorter and narrower in *A. laniger* . The talonid is narrower than the trigonid in *A. spenceri* , and an entoconid is present in both *A. laniger* and *A. longicaudatus* .

The m4 is similar to the m3 other than the following: the trigonid is wider than the talonid; the anterior cingulum is wider; distinct posterior buccal and lingual cingula are present on either side of the remnant talonid ridge; the hypoconulid notch is larger; the parastylid and protoconid are smaller; the paraconid and metaconid are slightly taller and equal in size. Only the cristid obliqua is present on the talonid, with no posthypocristid. The talonid is further reduced than all other *Antechinomys*; however, the m4 is overall smaller in *A. laniger* .

### 6.9 Distribution and Habitat

**FIGURE 10:**
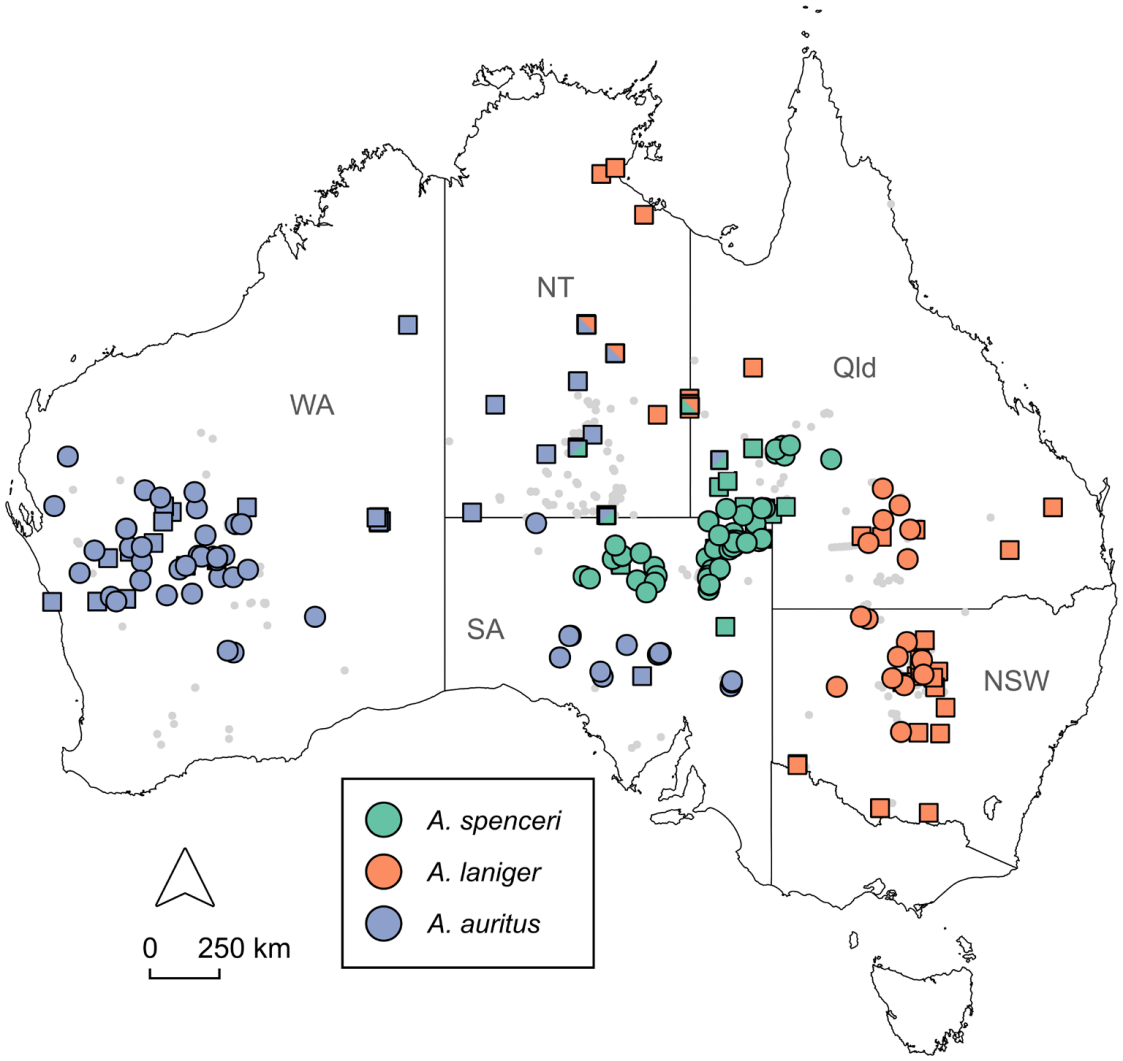
Map of known specimens of *Antechinomys spenceri*, *A. laniger* , and *A. auritus* sp. nov. based on genetic data (when available) and morphology. Circles show recent (< 50 years ago) specimens and squares show historical (> 50 years ago) specimens. Grey points show occurrence records sourced from the Atlas of Living Australia (ALA) and primary literature searches. Localities from which two species of kultarr have been recorded are shown by bicoloured points.

*Antechinomys auritus* appears to occur in low abundance across most of the western arid zone from the Simpson Desert in the east to the Carnarvon Basin in the west and prefers flat, open gibber and sandplain habitats that are patchily distributed between dune fields. In Western Australia, they are primarily known from the Murchison, Gascoyne, and Yalgoo Bioregions; however, they are also present in the Carnarvon Basin (Figure 10). There are very few records of *A. auritus* from the Gibson and Great Victoria Deserts, but this is likely due to the lack of scientific collection work carried out in these regions, and there is no evidence that extensive dunefields of the Great Victoria Desert act as a meaningful dispersal barrier for the species. *Antechinomys auritus* is known from several localities in the west of the Northern Territory; however, it appears most abundant in the Finke River basin and western Simpson Desert. There are also several records of *A. auritus* in the Gawler region of south‐central South Australia. While *A. auritus* and *A. spenceri* are sympatric in the Finke River basin, there are no records of *A. auritus* in the extensive stony plains of the Eyre Basin that appear to be the primary habitat of *A. spenceri* . There are specimens matching the morphology of *A. auritus* from Sandringham Station in the eastern Simpson Desert; however, the current and former distribution of *A. auritus* east of the Simpson Desert dunefields remains unclear and requires additional investigation.

## 7 Discussion

**FIGURE 11:**
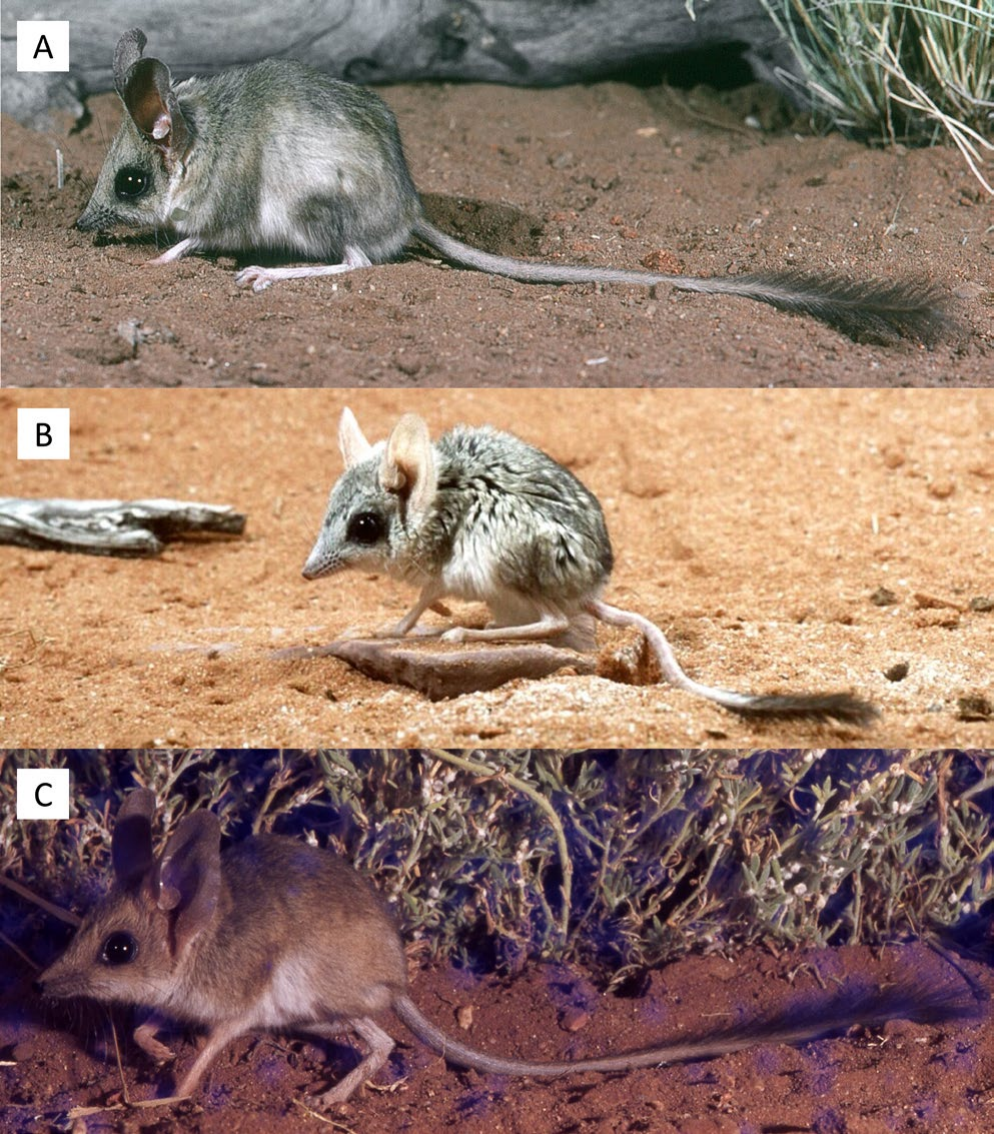
Live photographs of the three recognized kultarr species: A. *Antechinomys auritus* sp. nov. (photo credit Ken Johnson), B. *A. laniger* (photo credit Pat Woolley), C. *A. spenceri* (photo credit Ken Johnson). Photos approximately to scale.

In this study, we used genome‐wide SNP data, 12S mitochondrial data, linear skull‐shape data, and linear whole‐body measurements to demonstrate that the three clades of kultarr identified by Westerman et al. (2023) represent three distinct species: *Antechinomys laniger* , *A. spenceri* and *A. auritus* (Figure 11). However, our findings indicate that the distributions of these species differ substantially from those predicted by Westerman et al. (2023). Specifically, their ‘western’ clade actually extends across much of central Australia and their ‘central’ clade appears restricted to the extensive stony deserts of the Kati Thanda‐Lake Eyre basin. This highlights the importance of comprehensive geographic sampling and integrating both genetic and morphological data when resolving taxonomic uncertainty in widely distributed cryptic taxa.

Prior to this study, *Antechinomys* was considered to contain two species: *A. laniger* and *A. longicaudatus* (Westerman et al. 2023). With the description of *A. auritus* and resurrection of *A. spenceri* , *A. laniger* is now recognized as a species complex comprising three distinct taxa. These species add to the growing numbers of cryptic dasyurid taxa described since 2000, with that number now standing at 20 (AMTC 2024). Despite this progress, considerable taxonomic uncertainty remains at the species and genus level within the dasyurids (Westerman et al. 2016), and particularly within sminthopsins (Krajewski et al. 2012). We suggest that the taxonomic methods presented in the present study, namely: comprehensive geographic sampling; both phylogenetic and admixture analyses using genome‐wide SNP markers; and multiple forms of morphological data, serve as a benchmark for resolving the remaining taxonomic uncertainty in the group.

While the topology of our 12S and SNP phylogenetic trees differ, our SNP tree matches the topology of the multi‐gene tree generated by Westerman et al. (2023). Our support values are also quite low for our 12S tree. Given this, our data support the phylogenetic relationships proposed by Westerman et al. (2023), with *A. laniger* as sister to *A. spenceri* , and *A. auritus* as sister to both.

One sample, ABTC7522 from Betoota in southwest Queensland, appears to show nuclear‐mitochondrial discordance, with the SNP data from this sample matching *A. spenceri* and the 12S data matching *A. auritus* (Figure 3). Nuclear‐mitochondrial discordance is not uncommon in phylogenetic analyses and has been observed in another dasyurid genus, *Antechinus* (Mutton et al. 2019), and it may be a result of incomplete lineage sorting or mitochondrial introgression (Funk and Omland 2003). As we only identified one instance of discordance, it is unclear whether this is an anomaly or evidence of a more widespread pattern. Additional sampling in the putative sympatry zones of kultarr species is required to resolve this issue.

### 7.1 Morphological Convergence and Adaptation

With the resurrection of *A. spenceri* , Australia now has three small mammals recognized as gibber‐plains specialists that are sympatric in the Sturt Stony Desert: the kowari (Dasyuridae: *Dasyuroides byrnei* ) and the fawn hopping mouse (Muridae: *Notomys cervinus* ). All three species are highly adapted to arid environments, exhibiting elongated hindlimbs and large auditory bullae compared to their respective close relatives (Baker and Gynther 2023). Biomechanical studies of bipedal rodents with elongated hindlimbs suggest these adaptations optimize bursts of acceleration (generally leaps) as a predator avoidance strategy (Bradley‐Cronkwright et al. 2024; Moore, Cooper, et al. 2017; Moore, Rivera, and Biewener 2017). Hindlimb elongation has also been associated with arid adaptation in rodents (Moore et al. 2015), suggesting that such predator avoidance behaviors may be most effective in open environments (Berman 1985).

Similarly, enlarged auditory bullae have been linked to arid adaptation in several rodent (Alhajeri 2021a, 2021b; Alhajeri and Steppan 2018) and non‐rodent groups (Taylor et al. 2022), with predator avoidance widely hypothesized as the primary evolutionary driver (Lay 1972; Manoussaki et al. 2008; Nengovhela et al. 2019). Bullar size has also been experimentally associated with predator avoidance capacity in rodents (Lay 1974; Webster and Webster 1971, 1972). While kultarrs exhibit striking similarities to many arid‐zone rodents in hindlimb elongation and bullae inflation, it is unclear how well the rodent‐derived predator avoidance hypotheses apply to the kultarrs given the ecological (predator/prey vs. prey only in rodents) and biomechanical (quadrupedal vs. bipedal) differences (Jones et al. 2024). However, kultarr remains are highly abundant in many owl pellet deposits (e.g., Baynes and Baird 1992; Baynes and Jones 1993), meaning it is plausible that adaptation to avoiding predation, particularly against owls, has influenced the evolution of enlarged bullae and elongated hindlimbs in the kultarrs.

### 7.2 Conservation

*Antechinomys laniger* is currently listed as endangered in New South Wales (NSW DPIE 2024) but it is not listed federally (DCCEEW 2024). Following the taxonomic revision presented here, the conservation status of *A. laniger* will need to be reassessed to consider its updated and much reduced distribution. Historical specimens and occurrence records indicate that *A. laniger* was once widespread across much of semi‐arid eastern Australia (Figure 10); however, its modern range appears restricted to the Cobar Peneplain and north to the Mulga Lands IBRA7 region. There have been no records of *A. laniger* from the Top End since 1921 or from near Tobermorey Station, NT since 1968, and the only record from western Queensland was collected prior to 1940. This suggests that *A. laniger* may be extirpated from the northwestern portion of its former range, although targeted field surveys are needed to corroborate this. Additionally, the sparsity and age of *A. laniger* records from the Northern Territory, as well as the location uncertainty around many of the records, make determining the former distribution of *A. laniger* challenging. Habitat suitability modelling and examination of subfossil records will be required to better understand the former range of this species.

The conservation status of *A. spenceri* and *A. auritus* also requires assessment to determine any necessary management interventions, though there is no indication that either is under immediate extinction threat. Kultarrs naturally occur at very low population densities (Spencer 1896; Woolley 1984) and they are notoriously difficult to catch and study (Pavey et al. 2020). This presents a challenge when resolving extant distributions as records are so rarely obtained and can be separated by many hundreds of kilometres. However, comparisons between modern and historical records of both species suggest they have not experienced the substantial range contractions of *A. laniger*. Additional work on subfossil material from the Nullarbor, Great Victoria Desert, and Flinders Ranges, habitat suitability modelling, and more consistent and targeted field surveys are required to refine our understanding of the historical and current distribution of these species.

### 7.3 Future Work

While our systematic revision provides a substantial advancement to our understanding of kultarr diversity, additional specimens and genetic data are required to further refine species boundaries and distributions. Key regions requiring further sampling include the Great Western Woodlands in south‐west Western Australia, the Eyre Peninsula in South Australia, Finke River Basin in the Northern Territory and the Murray Darling Depression to the east of the Flinders Ranges. Additionally, a kultarr specimen held at the Natural History Museum in London, collected from Cedar Bay in Far‐North Queensland in 1893 (Lidicker 1965), presents an unresolved taxonomic issue. This specimen may represent an additional taxon, a substantial range extension for *A. laniger* , or be an erroneous record. Corroborating the species identity of this specimen will be important for our understanding of the diversity and evolution of *Antechinomys*.

Due to the difficulty in capturing kultarr and the scarcity of information about their ecology and behavior, current knowledge has been largely compiled from studies conducted across what is now three distinct species. Several studies have also been carried out in the zone of sympatry of *A. spenceri* and *A. auritus* , including field (Pavey et al. 2020; Woolley 1984) and laboratory population research (Old et al. 2020; Stannard et al. 2015, 2025; Stannard and Old 2014; Woolley 1984). Where possible, these datasets should be retroactively assigned to the correct kultarr species to clarify ecological differences between them. We recommend that all researchers working with kultarrs across Australia obtain genetic material to corroborate species identity. This will contribute to refining species distributions and improving our broader understanding of kultarr ecology.

## Author Contributions

**Cameron S. Dodd:** data curation (lead), formal analysis (lead), investigation (lead), writing – original draft (lead). **Linette S. Umbrello:** conceptualization (equal), funding acquisition (lead), methodology (equal), supervision (lead), writing – review and editing (equal). **Michael Westerman:** conceptualization (equal), methodology (supporting), writing – review and editing (equal). **Renee A. Catullo:** conceptualization (equal), methodology (equal), supervision (lead), writing – review and editing (equal). **Kenny J. Travouillon:** investigation (supporting), methodology (equal), supervision (equal), writing – review and editing (equal). **Andrew M. Baker:** funding acquisition (supporting), investigation (supporting), writing – review and editing (equal).

## Conflicts of Interest

The authors declare no conflicts of interest.

## Data Availability

The molecular sequence data generated in this study are available on Genbank (12S accession numbers PV089391‐PV089416) and the NCBI Sequence Read Archive (BioProject ID: PRJNA1228329). Data and code are available on Dryad (https://doi.org/10.5061/dryad.z612jm6p1).

## Appendix 1

**TABLE A1:** List of material examined in this study.

Reg. Number	Locality	Latitude	Longitude	Collected	Sex	Age	SNP	12S acc.	Form
AM M.48199	Mitchell Highway, NSW	−30.6757	146.4853	6/11/2021	M	A	y	PV089403	Spirit
AM M.51345	Yantabulla Swamp, NSW	−29.3226	144.4958	5/05/2010	U	A	y	PV089404	Spirit
AM M.38544	Oakleagh Homestead, NSW	−31.15	146.54	7/06/2002	M	A	y	OP805917	Spirit
ABTC7466	Koonchera Waterhole, SA	−26.5098	139.5746	U	F	U	y		None
ABTC7484	Wiluna, WA	−27.2399	120.926	U	U	U	y	PV089391	None
ABTC7522	Betoota, Qld	−25.6914	140.731	< 3/08/1985	U	U	y	PV089407	None
ABTC7523	Betoota, Qld	−25.6914	140.731	< 3/08/1985	U	U	y	PV089408	None
ABTC7527	Cobar, NSW	−31.4954	145.8343	< 3/08/1985	U	U	y		None
ABTC7528	Cobar, NSW	−31.4954	145.8343	< 3/08/1985	M	U	y	PV089405	None
QM A009431	Diamantina National Park, Qld	−23.6514	141.3713	00/10/2009	U	U	y	PV089409	None
QM A009432	Diamantina National Park, Qld	−23.6514	141.3713	00/10/2009	U	U	y	PV089410	None
SAMA M14025/ABTC26613	Dickinna Hill, SA	−26.8056	140.0042	9/05/1987	M	A	y		Spirit
SAMA M14026/ABTC26612	Dickinna Hill, SA	−26.8056	140.0042	9/05/1987	F	SA	y		Spirit
SAMA M16855/ABTC36452	Macumba Station, SA	−27.2622	135.5022	30/06/1992	F	A	y	PV089411	Spirit
SAMA M17010/ABTC33806	Walgidya Waterhole, SA	−27.3581	135.1586	10/12/1992	F	J	y		Spirit
SAMA M18140/ABTC35223	Mount Gason, SA	−27.4033	138.7008	26/04/1995	M	A	y		Spirit
SAMA M18141/ABTC35248	Mount Gason, SA	−27.4033	138.7008	26/04/1995	F	A	y		Spirit
SAMA M18169/ABTC35269	Kalladeina Bore, SA	−27.7219	139.025	27/04/1995	F	A	y		Spirit
SAMA M18188/ABTC35325	Dickinna Hill, SA	−26.7842	139.8739	3/05/1995	F	A	y		Spirit
SAMA M18189/ABTC35333	Dickinna Hill, SA	−26.7833	139.855	4/05/1995	F	A	y		Spirit
SAMA M18717/ABTC36394	Big Blyth Bore, SA	−28.0614	136.0472	4/03/1996	F	A	y		Spirit
SAMA M19029/ABTC65816	Wongyarra Waterhole, SA	−26.3717	140.3944	1/09/1996	M	A	y		Spirit
SAMA M19409/ABTC27372	Cowarie Homestead, SA	−27.3331	138.595	2/05/1997	M	A	y		Spirit
SAMA M19489/ABTC66256	Macumba Station, SA	−27.2569	135.505	31/08/1997	F	J	y		Spirit
QM JM19810	Wyandra, Qld	−27.3644	145.9611	27/06/2005	U	A	y	OP805918	Spirit
SAMA M21874/ABTC81154	Cadelga Waterhole, SA	−26.1978	140.3881	28/10/2002	F	A	y		Spirit
SAMA M21876/ABTC81157	Mudcarnie Waterhole, SA	−26.7728	140.5114	29/10/2002	F	A	y		Spirit
SAMA M22633/ABTC27493	Cliffton Hills Homestead, SA	−27.0175	138.8947	00/06/1998	F	A	y	PV089412	Skull
SAMA M23227/ABTC79279	Cliffton Hills Homestead, SA	−27.7172	138.7869	3/08/2000	M	A	y		Skull
SAMA M23604/ABTC102975	Peake Homestead, SA	−27.6222	136.6703	27/05/2005	M	A	y		Spirit
SAMA M23606/ABTC102977	Peake Homestead, SA	−27.6222	136.6703	29/05/2005	F	A	y		Spirit
SAMA M23607/ABTC102978	Peake Homestead, SA	−27.9194	136.7644	29/05/2005	M	A	y		Spirit
SAMA M23610/ABTC102981	Peake Homestead, SA	−28.1336	136.6939	31/05/2005	M	A	y		Spirit
SAMA M24966/ABTC139261	Donald's Well, SA	−26.1864	132.3478	22/03/2011	F	J	y	PV089392	Skull; Spirit
SAMA M25574/ABTC112645	Cadney Park, SA	−27.9228	134.0894	6/01/2011	F	A	y	OP808186	Spirit
SAMA M25583/ABTC139278	Olympic Dam, SA	−30.475	136.8875	12/12/2010	M	J	y		Spirit
SAMA M27351/ABTC141150	Cliffton Hills Homestead, SA	−27.0469	139.43	28/08/2015	F	A	y		Spirit
WAM M36269	Leinster, WA	−27.9519	120.7408	2003	M	A	y	PV089393	Spirit
WAM M46358	Yalgoo, WA	−28.6	116.7667	2003	F	A	y	PV089394	Spirit
WAM M48522	Milgun, WA	−25.1072	118.0078	1999	F	J	y	OP805921	Spirit
WAM M49653	Ora Banda, WA	−30.4256	121.2623	2002	M	SA	y		Skull
WAM M53977	Yalgoo, WA	−28.75	116.9667	2003	M	A	y		Spirit
WAM M56495	Lake Mason, WA	−27.6975	119.28	2005	F	A	y	OP805922	Spirit
WAM M57766	Weld Range, WA	−26.9833	117.4667	2007	M	A	y	PV089395	Spirit
WAM M57767	Weld Range, WA	−26.9833	117.4667	2007	M	A	y		Spirit
WAM M60766	Western Australia	NA	NA	2008	F	J	y	OP805924	Spirit
WAM M62126	Wooleen, WA	−27.0833	116.1667	2005	M	A	y		Spirit
WAM M62152	Ora Banda, WA	−30.3666	121.05	9/01/2006	M	SA	y	OP805925	Skull
WAM M64955	Boolardoo Station, WA	−27.8172	115.6233	2006	M	A	y	PV089396	Spirit
WAM M65259	Matuwa, WA	−26.216	121.343	2022	F	J	y	PV089397	Spirit
ABTC66258	Macumba Station, SA	−27.2569	135.505	7/08/1992	U	U	y		None
ABTC66259	Macumba Station, SA	−27.2569	135.505	7/08/1992	U	U	y		None
ABTC66260	Macumba Station, SA	−27.2569	135.505	7/08/1992	U	U	y		None
ABTC66261	Macumba Station, SA	−27.2569	135.505	7/08/1992	U	U	y	PV089413	None
ABTC66262	Macumba Station, SA	−27.2569	135.505	7/08/1992	U	U	y		None
ABTC66263	Macumba Station, SA	−27.2622	135.5022	7/08/1992	U	U	y		None
ABTC66402	Macumba Station, SA	−27.2622	135.5022	7/08/1992	U	U	y		None
ABTC117732	Olympic Dam, SA	−30.4978	136.8116	16/12/2005	F	U	y		None
ABTC118347	Borefield, SA	−30.4405	136.9167	U	F	U	y	PV089398	None
ABTC127916	Kingoonya, SA	−31.2197	134.7839	19/09/2012	F	U	y	PV089399	None
ABTC138050	Olympic Dam, SA	−30.4448	136.8699	U	U	U	y	PV089400	None
ABTC147788	Coober Pedy, SA	−29.8763	133.6182	14/11/2015	U	U	y	PV089401	None
ABTC150923	Mulka Station, SA	−28.2195	138.7106	3/08/2019	M	U	y		None
ABTC150924	Mulka Station, SA	−28.197	138.7074	3/08/2019	F	U	y		None
ABTC151613	Innamincka, SA	−26.8272	140.0614	21/09/2019	U	U	y		None
WAM TM1133	Nombinnie NR, NSW	−33.0318	145.7179	19/12/2018	M	A	y	OP805919	None
AM M.37162	Wanaaring, NSW	−29.25	144.25	23/10/2003	M	A		OP805916	Spirit
AM M.8428	Sandringham Station, Qld	−25	139.05	25/10/1963	M	A		PV089414	Skin; Skull
AM M.8461	Sandringham Station, Qld	−24.0667	139.05	25/10/1963	M	A		PV089415	Skin
JT_GVD01	Great Victoria Desert, WA	−29.2567	124.249	00/06/2024	U	A		PV089402	None
MVZ133202.2	El Trune Station, NSW	−31.1998	146.2926	27/05/1964	U	A		OP805920	Skin
QM JM19622	Quilpie, Qld	−26.8339	144.5276	20/06/2011	U	A		PV089406	Skin
QM JM819	Tobermorey, NT	−22.2833	137.97	00/09/1968	U	A			Skull
SAMA M18206/ABTC35188	Mungerannie, SA	−26.1239	138.6631	27/04/1995	M	A		KJ868097	Spirit
SAMA M18705/ABTC36361	Mussel Waterhole, SA	−28.4533	136.3906	28/02/1996	M	A		PV089416	Spirit
SAMA M3909	Yulabilla Station, Qld	−27.0667	149.7	1936	U	A			Skull; Spirit
AM M.8464	El Trune Station, NSW	−31.0833	146.4167	29/05/1964	M	A			Skull
AM M.8463	El Trune Station, NSW	−31.0833	146.4167	29/05/1964	F	A			Skull
QM JM1174	Tobermorey, NT	−22.1	137.97	U	M	A			Skull
QM JM1173	Tobermorey, NT	−22.1	137.97	U	M	A			Skull
SAMA M13912	Tobermorey, Qld	−22.4333	137.97	21/09/1968	F	A			Skull
NMV C6946	Deniliquin, NSW	−35.53	144.95	1883	M	A			Skull; Spirit
QM J11266	Cheepie, Qld	−26.6333	145.0167	U	M	A			Skull
WAM M53591	Grass Patch, WA	−33.2572	121.0961	2000	M	A			Skull; Spirit
QM JM820	Tobermorey, NT	−22.28	137.97	U	M	A			Skull
AM M.8465	El Trune Station, NSW	−31.0833	146.4167	30/05/1964	F	A			Skull
AM M.6954	Girilambone, NSW	−31.25	146.9	23/06/1944	M	A			Skull
WAM M20648	Toomey Hills, WA	−31.575	119.8667	1984	M	A			Skull; Spirit
QM JM2739	No data	NA	NA	U	U	A			Skull
WAM M20649	Toomey Hills, WA	−31.5833	119.85	1984	F	A			Skull; Spirit
NMV C2537	Tarlton Downs, NT	−22.63	136.8	1/01/1955	M	A			Skull; Spirit
AM M.8462	El Trune Station, NSW	−31.0833	146.4167	28/05/1964	M	A			Skull
SAMA M8404	Tobermorey, NT	−22.2667	137.97	21/09/1968	F	A			Skull
WAM M2230	Lake Grace, WA	−33.1	118.4667	1937	M	A			Skull
WAM M5079	Mount Talbot, WA	−26.1166	126.6667	1962	F	A			Skull
NMV C7924	Charlotte Waters, NT	−25.92	134.93	00/08/1895	U	A			Skull
NMV C6949	Charlotte Waters, NT	−25.92	134.93	00/04/1895	M	A			Skull; Spirit
WAM M2368	Lake Grace, WA	−33.1	118.4667	1939	F	A			Skull
QM JM1177	Betoota, Qld	−25.7	140.75	U	F	A			Skull
WAM M1546	Sturt Creek, WA	−19.6833	127.65	1931	U	SA			Skull
WAM M5776	Elder Creek, WA	−26.1166	126.5833	1/07/1963	M	A			Skull; Spirit
QM J23616	Sandringham Station, Qld	−24.05	139.0667	18/08/1971	M	A			Skull; Spirit
AM M.35822	Northern Territory	NA	NA	U	M	A			Skull
WAM M3815	Mileura Homestead, WA	−26.3666	117.3333	1975	F	A			Skull; Spirit
WAM M7737	Warbuton Range, WA	−26	126.5	1968	F	A			Skull; Spirit
SAMA M22202	Singer Dam, SA	−26.9611	139.5844	6/06/2003	U	A			Skull
AM M.4641	Rawlinna, WA	−31.0167	125.3333	8/12/1929	U	A			Skull
WAM M5774	Elder Creek, WA	−26.1166	126.5833	1/07/1963	F	A			Skull
SAMA M7935	Cootra East, SA	−29.5833	139.2833	9/04/1969	M	A			Skull
WAM M23674	Mileura Homestead, WA	−26.3666	117.3333	1986	M	A			Skull
WAM M8403	Meekatharra, WA	−27.3333	116.6667	U	U	A			Skull
AM M.8460	Sandringham Station, Qld	−24.05	139.0167	27/10/1963	M	A			Skull
WAM M23708	Mileura Homestead, WA	−26.3666	117.3333	1986	M	A			Skull
NMV C6936	Charlotte Waters, NT	−25.92	134.93	00/08/1895	M	A			Skull; Spirit
WAM M51079	New Springs Homestead, WA	−25.7	119.9167	1975	M	SA			Skull
WAM M5871	Warburton Mission, WA	−26.1333	126.5833	1963	F	A			Skull
WAM M6069	Warburton Mission, WA	−26.1333	126.5667	1964	M	A			Skull
QM JM3733	Diamantina Lakes, Qld	−23.7667	141.1333	00/12/1981	M	A			Skull; Spirit
WAM M5775	Elder Creek, WA	−26.1166	126.5833	1/07/1963	F	A			Skull; Spirit
WAM M23695	Mileura Homestead, WA	−26.3666	117.3333	1986	F	A			Skull
WAM M8403_1	Meekatharra, WA	−27.3333	116.6667	U	U	A			Skull
WAM M6082	Poona Mining Centre, WA	−27.125	117.4583	1964	M	A			Skull
SAMA M9485	Clifton Hills Station, SA	−27.0167	138.9	6/10/1973	M	A			Skull
WAM M6355	New Springs Homestead, WA	−25.8166	119	1964	F	A			Skull
WAM M7842	Peak Hill, WA	−25.6333	118.7167	1968	M	A			Skull; Spirit
WAM M23693	Mileura Homestead, WA	−26.3666	117.3333	1986	F	A			Skull
WAM M7738	Warburton Mission, WA	−26.0666	126.5833	1968	F	A			Skull; Spirit
NMV C7355	Sandringham Station, Qld	−24.05	139.07	24/08/1966	M	A			Skull
WAM M23710	Mileura Homestead, WA	−26.3666	117.3333	1986	M	A			Skull
SAMA M8259	Betoota, Qld	−25.8833	141	00/06/1969	M	A			Skull
SAMA M13911	Tobermorey, NT	−22.4333	137.97	21/09/1968	M	A			Skull
WAM M5886	Elder Creek, WA	−26.1166	126.5833	1963	F	A			Skull
SAMA M7525	Sandringham Station, Qld	−24.05	139.0667	10/09/1967	M	A			Skull
WAM M23734	Mileura Homestead, WA	−26.3666	117.3333	1986	M	A			Skull
WAM M15191	Cue, WA	−27.4333	117.9	1977	U	A			Skull
WAM M23688	Mileura Homestead, WA	−26.3666	117.3333	1986	F	A			Skull
WAM M6606	Warburton Mission, WA	−26.1666	126.5833	1965	M	J			Skull
SAMA M857	Roper River, NT	−14.7333	134.7333	16/05/1921	U	A			Skull
WAM M2750	Meekatharra, WA	−26.8333	118.3333	1948	U	J			Skull; Spirit
WAM M23687	Mileura Homestead, WA	−26.3666	117.3333	1986	F	A			Skull
SAMA M1805	Roper River, NT	−14.5333	135.25	00/12/1921	M	J			Skull
SAMA M28009	Challenger Gold Mine, SA	−29.8759	133.5814	9/04/2022	U	J			Skull
SAMA M1804	Roper River, NT	−14.5333	135.25	00/12/1921	M	A			Skull; Spirit
WAM M2860	Earaheedy Homestead, WA	−25.6666	121.75	1951	M	J			Skull
SAMA M7524	Wirraminna, SA	−31.2	136.2333	30/03/1968	M	A			Skull
SAMA M22957	Mungeranie Homestead, SA	−28.0167	138.6625	2/07/2002	M	A			Skull
SAMA M10922	Mount Sarah, SA	−26.9167	135.25	3/06/1981	M	A			Skull
SAMA M18133	Copper Hills, SA	−28	134.32	26/05/1992	M	A			Skull
SAMA M4079	Wankarei, NT	−25.8333	130	00/07/1933	M	A			Skull
SAMA M9891	Clifton Hills Station, SA	−26.75	139.5667	25/08/1975	F	A			Skull
SAMA M7523	Oodnadatta, SA	−27.4667	135.4333	15/06/1963	M	A			Skull
QM JM1176	Tobermorey, NT	−22.2833	137.97	U	F	J			Skull
SAMA M22203	Singer Dam, SA	−26.9617	139.5442	6/06/2003	U	J			Skull
SAMA M22961	Mungeranie Homestead, SA	−28.0167	138.6625	2/07/2002	U	J			Skull
WAM M23623	Peak Charles, WA	−32.9416	121.1	1986	M	A			Skull; Spirit
WAM M23624	Peak Charles, WA	−32.9416	121.1	1986	F	A			Skull; Spirit
WAM M7869	Barnabinmah Homestead, WA	−28.6666	117.3333	1968	U	A			Skull
QM JM1175	Tobermorey, NT	−22.2833	137.97	U	F	SA			Skull
QM J23615	Sandringham Station, Qld	−24.05	139.0667	11/11/1968	F	J			Skull; Spirit
SAMA M6202	Oodnadatta, SA	−27.55	135.45	23/04/1959	U	A			Skin
WAM M6620	Warburton Mission, WA	−26.1666	126.5833	1965	F	A			Spirit
WAM M4572	Mount Beazley, WA	−25.3444	118.5833	1977	U	A			Spirit
WAM M10013	Karalundi Mission, WA	−26.1333	118.6833	1973	M	A			Spirit
WAM M18161	Weebo Homestead	−27.9583	121.25	1979	M	A			Spirit
WAM M17615	Banjiwarn, WA	−27.7111	121.7917	1979	M	A			Spirit
WAM M7060	Wanjarri, WA	−27.4055	120.6417	1966	U	A			Spirit
WAM M23565	Wanjarri, WA	−27.3166	120.6667	1984	M	A			Spirit
WAM M9620	Mugga Mugga Hill, WA	−28.7555	116.2667	1972	M	A			Spirit
WAM M10526	Lake Mason Homestead, WA	−27.5833	119.5167	20/08/1973	M	A			Spirit
WAM M10012	Karalundi Mission, WA	−26.1333	118.6833	1973	M	A			Spirit
WAM M57990	Bulga Downs, WA	−28.4952	119.7419	2006	F	A			Spirit
WAM M23570	Wanjarri, WA	−27.3166	120.6667	1984	F	A			Spirit
WAM M23671	Mileura Homestead, WA	−26.3666	117.3333	1986	M	A			Spirit
WAM M23567	Wanjarri, WA	−27.3166	120.6667	1984	M	A			Spirit
WAM M23568	Wanjarri, WA	−27.3166	120.6667	1984	M	A			Spirit
WAM M23566	Wanjarri, WA	−27.3166	120.6667	1984	U	A			Spirit
WAM M23569	Wanjarri, WA	−27.3166	120.6667	1984	F	A			Spirit
WAM M23572	Wanjarri, WA	−27.3166	120.6667	1984	M	A			Spirit
WAM M37025	Marymia Hill, WA	−25.1666	119.8333	< 1991	M	A			Spirit
WAM M34476	Wiluna, WA	−26.5833	120.2333	24/08/1980	M	A			Spirit
WAM M23686	Mileura Homestead, WA	−26.3666	117.3333	1986	M	A			Spirit
WAM M40608	Wanjarri, WA	−27.4	120.65	00/05/1980	M	A			Spirit
WAM M40599	No data	NA	NA	2001	M	A			Spirit
WAM M25771	Yeelirrie Homestead, WA	−27.25	119.9833	1986	F	A			Spirit
WAM M23721	Mileura Homestead, WA	−26.3666	117.3333	1986	M	A			Spirit
WAM M27910	Youanmi Downs Homestead, WA	−28.55	118.8167	1986	F	A			Spirit
WAM M23736	Cobar, NSW	−31.5	145.8333	1986	M	SA			Spirit
WAM M48286	Mt Keith Station, WA	−27.2833	120.5167	18/08/1992	M	A			Spirit
WAM M41261	Lake Mason, WA	−27.6975	119.28	2004	M	A			Spirit
WAM M57341	Lorna Glen, WA	−26.2166	121.55	2007	F	A			Spirit
SAMA M3904	Central Australia	NA	NA	00/08/1902	F	A			Spirit
SAMA M682.001	Charlotte Waters, NT	−25.9167	134.9167	30/12/1915	M	A			Spirit
SAMA M3899	Central Australia	NA	NA	00/08/1902	M	A			Spirit
SAMA M3903	Central Australia	NA	NA	00/08/1902	M	A			Spirit
SAMA M682.002	Charlotte Waters, NT	−25.9167	134.9167	30/12/1915	F	A			Spirit
SAMA M3905	Central Australia	NA	NA	00/08/1902	M	A			Spirit
SAMA M3897	Central Australia	NA	NA	00/08/1902	M	A			Spirit
SAMA M3898	Central Australia	NA	NA	00/08/1902	M	A			Spirit
SAMA M682.003	Charlotte Waters, NT	−25.9167	134.9167	30/12/1915	U	A			Spirit
SAMA M3900	Central Australia	NA	NA	00/08/1902	M	A			Spirit
SAMA M233	Charlotte Waters, NT	−25.9167	134.9167	1936	M	A			Spirit
SAMA M6768	Vaughan Springs, NT	−22.3	130.85	00/05/1967	F	A			Spirit
SAMA M7765	Oodnadatta, SA	−27.55	135.45	20/07/1969	F	A			Spirit
SAMA M6387	Glengyle Station, Qld	−24.8	139.3667	14/06/1966	F	A			Spirit
SAMA M9223	Coorabulka Homestead, NT	−23.7333	140.3	00/09/1968	F	A			Spirit
SAMA M7165	Warburton Mission, WA	−25.9833	126.5833	00/05/1968	F	A			Spirit
SAMA M3912	Charlotte Waters, NT	−25.9167	134.9167	19/04/1905	F	A			Spirit
SAMA M3914	Charlotte Waters, NT	−25.9167	134.9167	1936	F	SA			Spirit
SAMA M13153	Pelican Waterhole, Qld	−25.6833	140.6167	1/09/1981	F	A			Spirit
SAMA M7988	Cordillo Downs, Qld	−26.7	140.6167	1/09/1970	F	A			Spirit
SAMA M9093	Clifton Hills Station, SA	−27.0167	138.9	6/10/1973	F	A			Spirit
SAMA M3917	Charlotte Waters, NT	−25.9167	134.9167	1936	M	A			Spirit
SAMA M9094	Clifton Hills Station, SA	−27.0167	138.9	6/10/1973	M	A			Spirit
SAMA M3907	Central Australia	NA	NA	00/08/1902	M	SA			Spirit
SAMA M9646	South East, Qld	NA	NA	13/07/1976	M	A			Spirit
SAMA M3911	Tennant Creek, NT	−19.65	134.1833	00/06/1905	M	A			Spirit
SAMA M3913	Charlotte Waters, NT	−25.9167	134.9167	1936	F	A			Spirit
SAMA M11454	Clifton Hills Station, SA	−26.75	139.6667	27/03/1984	M	A			Spirit
SAMA M9645	Cordillo Downs, SA	−26.7	140.6167	13/07/1976	M	A			Spirit
SAMA M3906	Central Australia	NA	NA	00/08/1902	M	A			Spirit
SAMA M16384	Kalladeina Bore, SA	−27.9	138.675	26/08/1990	F	A			Spirit
SAMA M16936	Pandie Pandie Homestead, SA	−26.8158	139.6033	4/08/1992	F	A			Spirit
SAMA M18266	Wire Creek Bore, SA	−27.2569	135.505	18/05/1995	M	A			Spirit
SAMA M14951	Erudina Homestead, SA	−31.53	139.47	17/10/1988	M	A			Spirit
SAMA M16860	Mulka, SA	−28.37	138.67	26/06/1992	M	A			Spirit
SAMA M14471	Mount Christie Siding, SA	−30.5881	133.2206	19/10/1987	M	A			Spirit
SAMA M16481	Mount Christie Siding, SA	−30.5881	133.2206	19/10/1987	F	A			Spirit
SAMA M14158	Mount Christie Siding, SA	−30.5881	133.2206	7/03/1987	F	A			Spirit
SAMA M18207	Mungerannie, SA	−27.9931	138.6411	30/04/1995	M	A			Spirit
SAMA M18204	Mungerannie, SA	−26.1239	138.6631	6/05/1995	M	A			Spirit
SAMA M17592	New Alton Downs, SA	−26.58	139.05	14/11/1994	M	A			Spirit
SAMA M22227	Curnamona, SA	−31.4119	139.5233	00/07/1990	F	A			Spirit
SAMA M24886	Tarcoola, SA	−29.8847	133.5669	7/11/2009	F	A			Spirit
SAMA M27972	Winnamincka, SA	−26.8272	140.0614	21/09/2019	F	A			Spirit
SAMA M24491	Tidnabucca Waterhole, SA	−27.1717	136.1833	14/03/2005	M	A			Spirit
SAMA M26298	Kingoonya, SA	−31.2197	134.7839	19/05/2012	M	A			Spirit
NMV C27890	Alice Springs, NT	−23.7	133.87	1916	M	A			Spirit
NMV C6948	Alice Springs, NT	−23.7	133.87	00/08/1895	M	A			Spirit
NMV C27891	Alice Springs, NT	−23.7	133.87	1916	M	A			Spirit
NMV C27892	Alice Springs, NT	−23.7	133.87	1916	M	A			Spirit
NMV C27883	Alice Springs, NT	−23.7	133.87	1916	F	A			Spirit
NMV C6953	Barrow Creek, NT	−21.53	133.88	6/06/1901	F	A			Spirit
NMV C6934	Barrow Creek, NT	−21.53	133.88	6/06/1901	M	A			Spirit
NMV C6957	Barrow Creek, NT	−21.53	133.88	6/06/1901	M	A			Spirit
NMV C6952	Barrow Creek, NT	−21.53	133.88	6/06/1901	M	A			Spirit
NMV C6955	Barrow Creek, NT	−21.53	133.88	6/06/1901	M	A			Spirit
NMV C6956	Barrow Creek, NT	−21.53	133.88	6/06/1901	F	A			Spirit
NMV C6958	Barrow Creek, NT	−21.53	133.88	6/06/1901	M	A			Spirit
NMV C6944	Borroloola, NT	−16.07	136.3	6/10/1911	M	A			Spirit
NMV C495	Charlotte Waters, NT	−25.92	134.93	19/07/1934	M	A			Spirit
NMV C493	Charlotte Waters, NT	−25.92	134.93	19/07/1934	F	A			Spirit
NMV C6937	Charlotte Waters, NT	−25.92	134.93	00/08/1895	M	A			Spirit
NMV C6935	Charlotte Waters, NT	−25.92	134.93	00/06/1896	M	A			Spirit
NMV C6939	Charlotte Waters, NT	−25.92	134.93	1895	F	A			Spirit
NMV C494	Charlotte Waters, NT	−25.92	134.93	19/07/1934	F	A			Spirit
NMV C6943	Deniliquin, NSW	−35.53	144.95	1883	M	A			Spirit
NMV C6942	Deniliquin, NSW	−35.53	144.95	1883	M	A			Spirit
NMV C6945	Deniliquin, NSW	−35.53	144.95	1883	M	A			Spirit
NMV C6947	Deniliquin, NSW	−35.53	144.95	1883	M	A			Spirit
NMV C6941	Deniliquin, NSW	−35.53	144.95	1883	M	A			Spirit
NMV C11087	Finke River, NT	−23.92	132.72	00/12/1929	F	A			Spirit
NMV C658	Tennant Creek, NT	−19.65	134.18	24/07/1901	M	A			Spirit
NMV C657	Tennant Creek, NT	−19.65	134.18	24/07/1901	M	A			Spirit
NMV C6950	Tennant Creek, NT	−19.65	134.18	24/07/1901	F	A			Spirit
NMV C660	Tennant Creek, NT	−19.65	134.18	24/07/1901	F	A			Spirit
NMV C659	Tennant Creek, NT	−19.65	134.18	24/07/1901	M	A			Spirit
WAM M18758	Yeelirrie Homestead, WA	−27.2819	120.0903	1980	U	A			Spirit
AM M.621	Walbundrie, NSW	−35.6833	146.7333	U	U	A			Spirit
AM A8248	Lachlan River, NSW	−33.0561	146.3566	U	U	A			Spirit
AM M.12789	Diamentina Lakes, Qld	−23.9667	141.45	11/03/1983	F	J			Spirit
AM M.7036	Girilambone, NSW	−31.25	146.9	29/06/1945	M	J			Spirit
NMV C6959	Barrow Creek, NT	−21.53	133.88	6/06/1901	F	A			Spirit
AM M.12792	Diamentina Lakes, Qld	−23.9	141.1333	11/04/1983	M	A			Spirit
AM M.12795	Diamentina Lakes, Qld	−23.9667	141.45	11/08/1983	M	A			Spirit
SAMA M16385	Mungerainie Gap, SA	−27.67	139.08	30/08/1990	M	A			Spirit
WAM M57342	Lorna Glen, WA	−26.2166	121.55	2007	M	A			Spirit
SAMA M3902	Central Australia	NA	NA	00/08/1902	F	A			Spirit
SAMA M16483	Mount Christie Siding, SA	−30.5881	133.2206	19/10/1987	M	A			Spirit
WAM M23684	Mileura Homestead, WA	−26.3666	117.3333	1986	M	A			Spirit
SAMA M11167	Mt. Sarah Homestead, SA	−26.9167	135.25	11/09/1975	F	A			Spirit
SAMA M17344	Kingoonya, SA	−30.18	135.67	19/03/1993	M	A			Spirit
WAM M23678	Mileura Homestead, WA	−26.3666	117.3333	1986	M	A			Spirit
WAM M57404	Williambury, WA	−23.9991	115.1903	2007	M	A			Spirit
AM M.49207	Sandringham Station, Qld	−24.0667	139.05	00/10/1968	M	SA			Spirit
WAM M23719	Mileura Homestead, WA	−26.3666	117.3333	1986	M	A			Spirit
WAM M23672	Mileura Homestead, WA	−26.3666	117.3333	1986	M	A			Spirit
WAM M23670	Mileura Homestead, WA	−26.3666	117.3333	1986	M	A			Spirit
SAMA M17343	Kingoonya, SA	−30.18	135.67	19/03/1993	F	A			Spirit
WAM M23713	Mileura Homestead, WA	−26.3666	117.3333	1986	F	SA			Spirit
AM M.8941	Sandringham Station, Qld	−24.05	139.0167	25/10/1963	F	A			Spirit
WAM M52122	Babyn Station, WA	−26.9666	117.9	1999	F	A			Spirit
SAMA M11166	Mt. Sarah Homestead, SA	−26.9167	135.25	12/12/1983	M	A			Spirit
AM M.9676	Sandringham Station, Qld	−24.0833	139.0667	29/10/1963	F	A			Spirit
WAM M40706	Mount Magnet, WA	−28.0666	117.85	1993	M	SA			Spirit
AM M.1806	Condobolin, NSW	−33.0833	147.15	5/12/1905	M	A			Spirit
NMV C6951	Barrow Creek, NT	−21.53	133.88	6/06/1901	M	A			Spirit
NMV C6933	Charlotte Waters, NT	−25.92	134.93	30/03/1905	M	A			Spirit
AM M.6863	Girilambone, NSW	−31.05	147.0833	8/12/1929	M	A			Spirit
AM M.6864	Byrock District, NSW	−30.6667	146.4	10/04/1943	F	A			Spirit
NMV C6954	Barrow Creek, NT	−21.53	133.88	6/06/1901	M	A			Spirit
WAM M36859	Lake Mason, WA	−27.5833	119.5167	1992	F	A			Spirit
AM M.7390	Miandetta, NSW	−31.5667	146.9667	27/05/1948	M	A			Spirit
AM M.8954	Tottenham, NSW	−32.2333	147.35	6/11/1966	M	A			Spirit
AM M.6880	Wyuna Downs, NSW	−30.0219	146.589	5/08/1944	M	J			Spirit
AM M.29237	Bourke, NSW	−30.0833	145.9333	23/10/1992	F	A			Spirit
WAM M6600	Warburton Mission, WA	−26.1666	126.5833	1965	M	A			Spirit
SAMA M27755	Mungeranie Homestead, SA	−28.2194	138.7106	7/03/2019	M	A			Spirit
SAMA M27754	Mungeranie Homestead, SA	−28.1969	138.7072	7/03/2019	F	A			Spirit
NMV C6932	Charlotte Waters, NT	−25.92	134.93	1901	F	A			Spirit
NMV C6940	Charlotte Waters, NT	−25.92	134.93	00/09/1895	M	A			Spirit
NMV C27884	Alice Springs, NT	−23.7	133.87	1916	M	U			Spirit
NMV C27887	Alice Springs, NT	−23.7	133.87	1916	F	A			Spirit
NHM 1847.12.4.5	Interior of NSW	−34.0822	141.9292	1847	M	A			Photo
NHM 1897.11.3.12	Charlotte Waters, NT	−25.92	134.93	1897	F	A			Photo
UJ Mam2552	Burnett River, Qld	−25.6651	151.2844	1892	U	A			CT‐scan
SAMA M683	Central Australia	NA	NA	00/08/1902	U	U			Spirit
WAM M6323	Warburton Mission, WA	−26.1333	126.5833	1964	U	J			Spirit
WAM M6619	Warburton Mission, WA	−26.1333	126.5833	U	U	J			Spirit
WAM M7739	Warburton Mission, WA	−26	126.5	1968	U	J			Spirit
WAM M23564	Wanjarri, WA	−27.3166	120.6667	1984	F	SA			Spirit
WAM M23571	Wanjarri, WA	−27.3166	120.6667	1984	F	SA			Spirit
WAM M48180	Little Sandy Desert, WA	NA	NA	U	U	J			Spirit
WAM M48057	Meedo Homestead, WA	−25.6228	114.6942	9/01/2006	U	J			Spirit
AM M.25513	Bourke, NSW	−30.5667	145.6	1991	M	J			Spirit
AM M.34567	Wilcannia, NSW	−31.55	143.3667	1999	M	J			Spirit
AM M.9678	Betoota, Qld	−25.65	139.9833	11/04/1963	M	J			Spirit
AM M.11400	Cobar, NSW	−31.2667	145.4	1980	M	SA			Spirit
AM M.8955	Cobar, NSW	−31.4	145.7667	16/01/1969	M	J			Spirit
AM M.7330	Girilambone, NSW	−31.25	146.9	1948	M	J			Spirit
SAMA M14144	Erudina Station, SA	−31.3667	139.525	00/02/1986	U	J			Spirit
SAMA M16482	Mount Christie Siding, SA	−30.5881	133.2206	19/10/1987	U	A			Spirit
SAMA M1806	Roper River, NT	−14.5333	135.25	00/12/1921	U	J			Spirit
SAMA M23162	Palkagee, SA	−33.5542	135.8556	13/02/2005	M	J			Spirit
SAMA M24492	Tidnabucca Waterhole, SA	−27.1719	136.1778	14/03/2005	U	J			Spirit
SAMA M2582	Geraldton, WA	−28.7667	114.6167	1929	U	J			Spirit
SAMA M27155	Kingoonya, SA	−31.0556	134.7036	2014	F	J			Spirit
SAMA M3654	Walthayalkanna, SA	−27.1	132.4333	00/02/1933	U	J			Spirit
SAMA M3893	No data	NA	NA	1936	U	J			Spirit
SAMA M3901	Central Australia	NA	NA	00/08/1902	U	J			Spirit
SAMA M3915	Charlotte Waters, NT	−25.9167	134.9167	1936	U	J			Spirit
SAMA M3918	Charlotte Waters, NT	−25.9167	134.9167	1936	U	J			Spirit
QM JM23615	Sandringham Station, Qld	−24.05	139.0667	U	F	J			Skull; Spirit
NMV C12268	Wentworth, NSW	−34.12	141.92	03/10/1857	M	A			Mount
QM J2739	No data	NA	NA	U	U	A			Spirit
QM JM3732	Diamentina Lakes, Qld	−23.7667	141.1333	00/12/1981	F	J			Spirit
QM JM3897	Charleville, Qld	−25.6	145.6667	00/04/1982	U	A			Spirit
QM JM4342	Durrie Station, Qld	−25.9333	139.9333	17/09/1982	M	A			Spirit
QM JM4446	Mt Leonard, Qld	−25.7	140.6167	12/04/1984	M	A			Spirit
QM JM5230	Birdsville, Qld	−25.7167	139.3167	29/07/1985	M	A			Spirit
QM J6516	Cloncurry, Qld	−21.0833	140.3	< 1940	U	A			Spirit
QM J8437	Charleville, Qld	−26.4	146.25	< 1955	U	A			Spirit
QM JM11124	Mt Windsor, Qld	−23.6333	141.65	1/07/1995	U	A			Spirit
QM JM12922	Alice Springs, NT	−23.2833	134.4167	4/08/1975	M	A			Spirit
QM JM13407	Mt Calder, Qld	−25.05	145.05	23/11/1990	U	J			Spirit
QM J15333	Windorah, Qld	−25.65	141.5	18/07/1968	U	A			Spirit
QM JM18397	Noonbah Station, Qld	−24.0889	143.1583	15/04/1997	F	A			Spirit
QM J23541	Tobermorey, NT	−22.2833	137.97	20/09/1968	M	A			Spirit
QM J23542	Tobermorey, NT	−22.2833	137.97	20/09/1968	F	A			Spirit
QM JM5020	Mariala NP, Qld	−26.0833	145.0667	16/09/1984	U	A			Spirit
QM JM11581	Charleville, Qld	−26.3941	146.0541	13/02/1994	U	A			Spirit
QM JM18082	Quilpie, Qld	−26.61	144.27	U	U	A			Spirit
QM JM19083	Quilpie, Qld	−26.61	144.27	U	U	A			Spirit
QM JM19084	Quilpie, Qld	−26.61	144.27	U	U	A			Spirit
NMV C496	Charlotte Waters, NT	−25.92	134.93	< 1934	M	J			Spirit
NMV C6938	Central Australia	NA	NA	1927	M	J			Spirit
NMV C25068	Alice Springs, NT	−23.7	133.87	U	F	J			Spirit
NMV C25069	Alice Springs, NT	−23.7	133.87	U	F	J			Spirit
NMV C27885	No data	NA	NA	U	M	J			Spirit
NMV C27886	No data	NA	NA	1916	M	A			Spirit
NMV C27888	No data	NA	NA	U	F	A			Spirit
NMV C27889	Alice Springs, NT	−23.7	133.87	1916	F	J			Spirit
ABTC7644^a^	Young Range, WA	−25.0416	124.9917	28/06/1981	U	U	y		None
MJ_M01^a^	Lofty Range, WA	−24.209	119.3861	27/03/2023	U	U	y		None
MJ_M02^a^	Lofty Range, WA	−24.1936	118.9992	5/09/2023	U	U	y		None
WAM M24531^a^	Young Range, WA	−25.0416	124.9917	2/07/1981	F	A	y	AF088972	Spirit

Abbreviations: Species (*Antechinomys*), museum/collection registration number (ABTC, Australian Biological Tissue Collection (housed at SAMA); AMS, Australian Museum Sydney; MVZ, Museum of Vertebrate Zoology Berkeley, California, USA; NHM, National History Museum, London, UK; NMV, National Museum of Victoria; QM, Queensland Museum; SAMA, South Australian Museum; UJ, University of Jena, Germany; WAM, Western Australian Museum), Locality (NSW, New South Wales; NT, Northern Territory; Qld, Queensland; SA, South Australia; WA, Western Australia), Sex (F, female; M, male; U, undetermined or indeterminable), Age (A, age; J, juvenile; SA, subadult).

^a^ *Antechinomys longicaudatus* genetic material used in this study.

**TABLE A2:** Downstream filtering pipeline for the four SNP datasets used in this study.

Pipeline and functions		Datasets and parameters				
Filtering Rationale	Package: Function()	Parameters	Phylogenetics	Population Genetics	Central Admixture	Eastern Admixture
Remove low quality samples	SNPfiltR: missing_by_sample()	cutoff	0.99	0.96	0.6	0.6
Remove low coverage sequences	SNPfiltR: hard filter()	depth	10	10	10	10
		genotype_quality	30	30	30	30
Remove SNPs with high heterozygosity	custom code	max_heterozygosity	0.5	0.5	0.5	0.5
Remove SNPs with highly discordant heterozygote frequency	SNPfiltR: filter_allele_balance()	min_value	0.25	0.25	0.25	0.25
		max_value	0.75	0.75	0.75	0.75
Remove paralogous sequences	SNPfiltR: max_depth()	max_depth	150	150	150	150
Remove invariant sites	SNPfiltR: min_mac()	min_mac	1	1	1	1
Remove SNPs with high missing rate	SNPfiltR: missing_by_snp()	cutoff	0.7	0.7	0.9	0.95
Remove SNPs with low reproducibility	custom code	min_reproducibility	0.97	0.97	NA	NA
Remove technical replicate samples	dartR.base: gl.keep.ind()	NA				
Remove SNPs with very rare minor alleles	dartR.base: gl.filter.maf()	threshold	2	2	2	2
Remove SNPs with high missing rate	dartR.base: gl.filter.callrate()	threshold	0.8	0.9	1	1
Remove secondaries	custom code	NA				

*Note:* SNPs were initially called from raw sequence data using the *Stacks2* pipeline (Rochette et al. 2019) as implemented using the *Galaxy* (v.24.1.3.dev0) bioinformatics platform (The Galaxy Community 2024). Full details of upstream filtering steps are shown in the text. Subsets of samples used in each filtering pathway are shown in this table.

**TABLE A3:** List of samples used in each filtering dataset (see Section 2 and Table 1).

Species (clade)	ID	Phylogenetics Dataset	Population Genetics Dataset	Central Admixture Dataset	Eastern Admixture Dataset
*A. laniger* (east)	ABTC7527	y	y		y
*A. laniger* (east)	ABTC7528	y	y		y
*A. laniger* (east)	AM M.37162	y	y		y
*A. laniger* (east)	AM M.38544	y	y		y
*A. laniger* (east)	AM M.48199	y	y		y
*A. laniger* (east)	AM M.51345	y	y		y
*A. laniger* (east)	QM JM19810	y	y		y
*A. laniger* (east)	WAM TM1133	y	y		y
*A. laniger* (central)	ABTC151613	y	y		y
*A. laniger* (central)	ABTC26612	y	y		y
*A. laniger* (central)	ABTC65816	y	y		y
*A. laniger* (central)	ABTC7523	y	y		y
*A. laniger* (central)	ABTC81154	y	y		y
*A. laniger* (central)	ABTC81157	y	y		y
*A. laniger* (central)	QM A009431	y	y		y
*A. laniger* (central)	QM A009432	y	y		y
*A. laniger* (central)	ABTC33806	y	y	y	
*A. laniger* (central)	ABTC36452	y	y	y	
*A. laniger* (central)	ABTC66263	y	y	y	
*A. laniger* (central)	ABTC66402	y	y	y	
*A. laniger* (central)	SAMA M18717	y	y	y	
*A. laniger* (central)	SAMA M19489	y	y	y	
*A. laniger* (central)	SAMA M23604	y	y	y	
*A. laniger* (central)	SAMA M23606	y	y	y	
*A. laniger* (central)	SAMA M23607	y	y	y	
*A. laniger* (central)	SAMA M23610	y	y	y	
*A. laniger* (central)	SAMA M25574	y	y	y	
*A. laniger* (west)	ABTC127916	y	y	y	
*A. laniger* (west)	ABTC138050	y	y	y	
*A. laniger* (west)	ABTC139261	y	y	y	
*A. laniger* (west)	ABTC117732	y	y	y	
*A. laniger* (west)	ABTC147788	y	y	y	
*A. laniger* (west)	SAMA M25583	y	y	y	
*A. laniger* (west)	ABTC118347	y	y	y	
*A. laniger* (central)	ABTC141150_Replicate	y	y		
*A. laniger* (central)	ABTC141150	y	y		
*A. laniger* (central)	ABTC150923	y	y		
*A. laniger* (central)	ABTC150924	y	y		
*A. laniger* (central)	ABTC151613_Replicate	y	y		
*A. laniger* (central)	ABTC26612_Replicate	y	y		
*A. laniger* (central)	ABTC26613	y	y		
*A. laniger* (central)	ABTC35325	y	y		
*A. laniger* (central)	ABTC35333_Replicate	y	y		
*A. laniger* (central)	ABTC35333	y	y		
*A. laniger* (central)	ABTC36452_Replicate	y	y		
*A. laniger* (central)	ABTC66258	y	y		
*A. laniger* (central)	ABTC66259	y	y		
*A. laniger* (central)	ABTC66260	y	y		
*A. laniger* (central)	ABTC66261	y	y		
*A. laniger* (central)	ABTC66262	y	y		
*A. laniger* (central)	ABTC7466_Replicate	y	y		
*A. laniger* (central)	ABTC7466	y	y		
*A. laniger* (central)	ABTC7522	y	y		
*A. laniger* (central)	ABTC7523_Replicate	y	y		
*A. laniger* (central)	SAMA M18140_Replicate	y	y		
*A. laniger* (central)	SAMA M18140	y	y		
*A. laniger* (central)	SAMA M18141_Replicate	y	y		
*A. laniger* (central)	SAMA M18141	y	y		
*A. laniger* (central)	SAMA M18169_Replicate	y	y		
*A. laniger* (central)	SAMA M18169	y	y		
*A. laniger* (central)	SAMA M18207	y	y		
*A. laniger* (central)	SAMA M19409_Replicate	y	y		
*A. laniger* (central)	SAMA M19409	y	y		
*A. laniger* (central)	SAMAM22633	y	y		
*A. laniger* (central)	SAMA M23227_Replicate	y	y		
*A. laniger* (central)	SAMA M23227	y	y		
*A. laniger* (west)	ABTC118347_Replicate	y	y		
*A. laniger* (west)	ABTC127916_Replicate	y	y		
*A. laniger* (west)	ABTC7484	y	y		
*A. laniger* (west)	WAM M36269	y	y		
*A. laniger* (west)	WAM M46358	y	y		
*A. laniger* (west)	WAM M48522	y	y		
*A. laniger* (west)	WAM M49653	y	y		
*A. laniger* (west)	WAM M53977	y	y		
*A. laniger* (west)	WAM M56495	y	y		
*A. laniger* (west)	WAM M57766	y	y		
*A. laniger* (west)	WAM M57767	y	y		
*A. laniger* (west)	WAM M60766	y	y		
*A. laniger* (west)	WAM M62126	y	y		
*A. laniger* (west)	WAM M62152	y	y		
*A. laniger* (west)	WAM M64955	y	y		
*A. laniger* (west)	WAM M65259	y	y		
*A. longicaudatus*	ABTC7644	y			
*A. longicaudatus*	MJ_M01	y			
*A. longicaudatus*	MJ_M02	y			
*A. longicaudatus*	WAM M24531	y			

**TABLE A4:** Summary of statistical tests performed on the external measurements dataset.

Kruskal–Wallis tests					Post hoc pairwise Wilcoxon tests			
Character	*n*	df	*H*	*p*	Comparison (*n*)	*W*	*p*	sig.
Head‐body length	175	2	46.61	*p* < 0.0001	East (23) vs. West (81)	174.5	< 0.0001	***
					East (23) vs. Central (71)	98.5	< 0.0001	***
					West (81) vs. Central (71)	2274.0	0.026	*
Tail length	168	2	68.48	*p* < 0.0001	East (27) vs. West (72)	438.0	< 0.0001	***
					East (27) vs. Central (69)	100.5	< 0.0001	***
					West (72) vs. Central (69)	972.0	< 0.0001	***
Ear length	198	2	64.39	*p* < 0.0001	East (38) vs. West (80)	217.0	< 0.0001	***
					East (38) vs. Central (79)	309.5	< 0.0001	***
					West (80) vs. Central (79)	3617.0	0.116	ns
Brush length	164	2	41.45	*p* < 0.0001	East (27) vs. West (69)	305.5	< 0.0001	***
					East (27) vs. Central (68)	181.0	< 0.0001	***
					West (69) vs. Central (68)	1799.5	0.018	*
Hindfoot length	209	2	132.22	*p* < 0.0001	East (39) vs. West (87)	540.0	< 0.0001	***
					East (39) vs. Central (81)	0.0	< 0.0001	***
					West (87) vs. Central (81)	625.0	< 0.0001	***
Forearm length	206	2	84.53	*p* < 0.0001	East (38) vs. West (85)	264.0	< 0.0001	***
					East (38) vs. Central (81)	78.0	< 0.0001	***
					West (85) vs. Central (81)	2262.0	0.0001	**
Head length	168	2	87.95	*p* < 0.0001	East (21) vs. West (78)	99.0	< 0.0001	***
					East (21) vs. Central (69)	1.0	< 0.0001	****
					West (78) vs. Central (69)	875.0	< 0.0001	****
Head width	167	2	60.67	*p* < 0.0001	East (22) vs. West (75)	206.0	< 0.0001	****
					East (22) vs. Central (70)	63.5	< 0.0001	****
					West (75) vs. Central (70)	1348.0	< 0.0001	****

*Note:* For each, a Kruskal–Wallis test was performed to detect differences between groups before post hoc pairwise Wilcoxon tests were used to compare each clade. Significance thresholds for the Wilcoxon tests were adjusted using a Benjamini‐Hochberg multiple test correction; however, this did not change the significance of any test.

**TABLE A5:** Summary of all kultarr skull and body measurements by clade and sex.

Character		East clade		West clade		Central clade	
		F	M	F	M	F	M
Skull							
bcl	*n*	5	7	7	7	3	15
	$\bar{x}\pm \sigma$	22.4 ± 0.2	23.2 ± 0.9	25.3 ± 0.9	26.0 ± 0.9	27.6 ± 0.5	27.4 ± 0.9
	Range	22.1–22.7	21.8–24.1	24.5–26.7	24.7–27.0	27.1–28.1	26.2–29.0
onl	*n*	4	8	7	7	3	15
	$\bar{x}\pm \sigma$	24.1 ± 0.2	25.0 ± 0.7	26.9 ± 0.8	27.5 ± 0.8	29.0 ± 0.7	28.8 ± 0.6
	Range	23.9–24.4	23.8–25.8	26.1–28.0	26.2–28.4	28.3–29.5	27.9–29.8
ZW	*n*	5	8	9	6	3	14
	$\bar{x}\pm \sigma$	13.5 ± 0.5	13.8 ± 0.5	14.8 ± 0.6	15.0 ± 0.9	16.1 ± 0.6	15.5 ± 0.6
	Range	12.9–14.2	13.3–14.7	13.9–15.6	14.0–16.2	15.6–16.7	14.1–16.3
HT	*n*	5	9	9	8	3	15
	$\bar{x}\pm \sigma$	9.3 ± 0.2	9.6 ± 0.4	10.8 ± 0.3	11.0 ± 0.5	11.1 ± 0.5	11.0 ± 0.3
	Range	9.0–9.5	9.2–10.1	10.1–11.1	9.8–11.5	10.8–11.8	10.3–11.4
HT.B	*n*	5	9	9	8	3	15
	$\bar{x}\pm \sigma$	7.0 ± 0.3	7.1 ± 0.3	7.9 ± 0.4	7.8 ± 0.6	8.1 ± 0.3	7.9 ± 0.2
	Range	6.7–7.4	6.8–7.6	7.4–8.3	6.5–8.4	7.9–8.4	7.5–8.3
lw	*n*	5	9	7	6	3	15
	$\bar{x}\pm \sigma$	8.9 ± 0.2	9.2 ± 0.5	9.9 ± 0.4	10.2 ± 0.7	10.7 ± 0.5	10.6 ± 0.5
	Range	8.7–9.1	8.6–10	9.4–10.3	9.4–11.2	10.4–11.3	9.5–11.6
nl	*n*	5	9	8	8	3	15
	$\bar{x}\pm \sigma$	8.3 ± 0.4	8.7 ± 0.4	9.4 ± 0.5	9.6 ± 0.5	11.1 ± 0.4	10.8 ± 0.6
	Range	7.7–8.7	8.2–9.3	8.7–10.3	8.9–10.4	10.7–11.5	9.8–11.6
PML	*n*	5	8	8	8	3	15
	$\bar{x}\pm \sigma$	6.8 ± 0.3	6.8 ± 0.8	7.3 ± 0.8	7.7 ± 0.6	8.2 ± 0.3	8.5 ± 0.5
	Range	6.4–7.2	5.2–7.5	6.2–8.3	6.8–8.3	7.9–8.4	7.6–9.4
ppw	*n*	5	9	9	7	3	15
	$\bar{x}\pm \sigma$	6.2 ± 0.5	6.5 ± 0.3	6.9 ± 0.3	7.0 ± 0.6	7.3 ± 0.4	7.5 ± 0.6
	Range	5.7–6.9	6.1–6.9	6.3–7.3	6.1–7.9	7.0–7.8	6.2–8.4
IOW	*n*	5	9	9	8	3	15
	$\bar{x}\pm \sigma$	5.2 ± 0.3	5.2 ± 0.2	5.8 ± 0.2	5.8 ± 0.4	5.6 ± 0.2	5.9 ± 0.4
	Range	4.9–5.6	5.0–5.5	5.5–6.0	5.3–6.5	5.5–5.8	5.4–6.7
NWR	*n*	5	9	9	7	3	15
	$\bar{x}\pm \sigma$	2.2 ± 0.4	2.3 ± 0.2	2.3 ± 0.3	2.6 ± 0.3	2.4 ± 0.5	2.5 ± 0.2
	Range	1.7–2.7	1.8–2.5	1.8–2.7	2.1–3.1	1.9–2.7	2.1–3.0
anw	*n*	5	9	8	8	3	15
	$\bar{x}\pm \sigma$	1.2 ± 0.1	1.3 ± 0.1	1.4 ± 0.1	1.4 ± 0.2	1.6 ± 0.3	1.5 ± 0.1
	Range	1.1–1.3	1.1–1.4	1.3–1.5	1.2–1.7	1.3–1.9	1.3–1.7
OBW	*n*	5	9	9	7	3	15
	$\bar{x}\pm \sigma$	9.9 ± 0.4	10.2 ± 0.4	11.5 ± 0.4	11.5 ± 0.5	12.1 ± 0.4	11.8 ± 0.4
	Range	9.3–10.3	9.6–10.7	10.8–12.2	10.8–12.3	11.9–12.6	11.3–12.7
MW	*N*	5	9	9	7	3	15
	$\bar{x}\pm \sigma$	10.7 ± 0.3	10.6 ± 0.5	11.9 ± 0.5	12.3 ± 0.4	12.3 ± 0.2	12.3 ± 0.4
	Range	10.2–11.0	9.7–11.4	11.2–12.6	11.8–13	12.0–12.5	11.5–13.0
BPL	*n*	5	9	9	7	3	15
	$\bar{x}\pm \sigma$	6.0 ± 0.2	6.1 ± 0.3	7.9 ± 0.2	7.9 ± 0.2	7.7 ± 0.1	7.8 ± 0.2
	Range	5.7–6.3	5.8–6.8	7.6–8.3	7.8–8.1	7.6–7.8	7.2–8.1
BOL	*n*	5	9	8	7	3	15
	$\bar{x}\pm \sigma$	3.4 ± 0.3	3.4 ± 0.2	3.8 ± 0.3	4.0 ± 0.2	3.7 ± 0.2	4.1 ± 0.3
	Range	3.0–3.8	3.2–3.9	3.3–4.5	3.5–4.2	3.5–3.8	3.7–4.9
IBW	*n*	5	9	9	7	3	15
	$\bar{x}\pm \sigma$	2.6 ± 0.2	2.7 ± 0.3	1.8 ± 0.3	2.4 ± 0.4	2.6 ± 0.2	2.8 ± 0.3
	Range	2.4–2.8	2.3–3.0	1.5–2.3	1.9–2.9	2.5–2.8	2.2–3.2
jl	*n*	5	9	8	7	3	15
	$\bar{x}\pm \sigma$	17.3 ± 0.3	18.1 ± 0.7	19.5 ± 1.3	19.8 ± 1	20.9 ± 1.4	21.0 ± 0.9
	Range	17.0–17.8	17.2–19.3	16.6–20.8	18.2–21.0	19.3–22.0	19.5–22.8
tr	*n*	5	9	9	7	3	13
	$\bar{x}\pm \sigma$	11.7 ± 0.2	11.9 ± 0.4	12.5 ± 0.2	12.8 ± 0.4	13.7 ± 0.5	13.9 ± 0.4
	Range	11.5–11.9	11.3–12.6	12.3–12.9	12.3–13.3	13.3–14.3	13.2–14.6
mr	*n*	5	9	9	7	3	15
	$\bar{x}\pm \sigma$	5.8 ± 0.1	5.8 ± 0.1	6.1 ± 0.2	6.2 ± 0.1	6.4 ± 0.2	6.6 ± 0.2
	Range	5.8–6.0	5.7–6.0	5.8–6.5	6.0–6.3	6.2–6.6	6.2–7.0
PL	*n*	5	6	8	8	2	14
	$\bar{x}\pm \sigma$	12.5 ± 0.6	12.7 ± 0.6	13.7 ± 0.4	14.0 ± 0.4	NA	15.1 ± 0.5
	Range	11.6–13.4	11.9–13.6	13.2–14.5	13.4–14.5	15.3–15.5	14.5–15.7
APV	*n*	4	9	5	7	3	14
	$\bar{x}\pm \sigma$	3.7 ± 0.2	3.8 ± 0.3	4.0 ± 0.2	4.0 ± 0.3	4.9 ± 0.1	4.5 ± 0.3
	Range	3.4–4.0	3.3–4.3	3.7–4.1	3.6–4.4	4.9–5.0	4.0–5.0
IPV	*n*	4	9	8	8	3	15
	$\bar{x}\pm \sigma$	2.0 ± 0.1	2.1 ± 0.4	2.2 ± 0.3	2.0 ± 0.4	2.3 ± 0.1	2.4 ± 0.5
	Range	1.9–2.1	1.7–2.9	1.8–2.7	1.6–2.5	2.2–2.4	1.8–3.9
PPV	*n*	4	5	7	7	2	15
	$\bar{x}\pm \sigma$	3.5 ± 0.2	3.7 ± 0.6	3.9 ± 0.3	4.3 ± 0.4	NA	4.39 ± 0.4
	Range	3.3–3.7	2.9–4.6	3.4–4.3	3.8–4.9	4.7–4.7	3.3–4.9
R.LM3	*n*	5	9	9	6	3	15
	$\bar{x}\pm \sigma$	9.5 ± 0.3	9.8 ± 0.4	10.5 ± 0.3	10.5 ± 0.2	10.9 ± 0.3	10.8 ± 0.5
	Range	9.0–9.8	9.1–10.4	10.1–10.8	10.2–10.9	10.6–11.1	9.6–11.4
R.LM2	*n*	5	9	9	6	3	15
	$\bar{x}\pm \sigma$	8.0 ± 0.2	8.2 ± 0.3	9.0 ± 0.3	8.9 ± 0.4	9.5 ± 0.3	9.3 ± 0.3
	Range	7.8–8.3	7.8–8.6	8.5–9.3	8.4–9.4	9.2–9.7	8.5–9.8
R.LM1	*n*	5	9	9	6	3	15
	$\bar{x}\pm \sigma$	7.3 ± 0.2	7.4 ± 0.3	8.1 ± 0.3	8.2 ± 0.5	8.6 ± 0.4	8.4 ± 0.3
	Range	7.1–7.5	7.0–7.9	7.7–8.4	7.5–8.6	8.2–9.0	7.6–8.9
R.LM1T	*n*	5	9	9	6	3	15
	$\bar{x}\pm \sigma$	6.2 ± 0.3	6.3 ± 0.3	7.1 ± 0.4	7.1 ± 0.5	7.3 ± 0.1	7.1 ± 0.2
	Range	5.9–6.6	5.9–6.7	6.6–7.7	6.3–7.5	7.1–7.3	6.5–7.4
R.LC1	*n*	5	9	9	7	3	15
	$\bar{x}\pm \sigma$	3.6 ± 0.1	3.7 ± 0.2	4.1 ± 0.2	4.1 ± 0.3	4.3 ± 0.3	4.3 ± 0.2
	Range	3.4–3.7	3.4–4.0	3.9–4.4	3.6–4.3	4.1–4.6	3.8–4.7
SWR.LC1B	*n*	5	9	9	7	3	15
	$\bar{x}\pm \sigma$	3.2 ± 0.1	3.3 ± 0.1	3.6 ± 0.1	3.6 ± 0.3	3.7 ± 0.1	3.7 ± 0.2
	Range	3.1–3.3	3.0–3.5	3.5–3.9	3.2–3.9	3.6–3.9	3.2–4.0
UML	*n*	5	9	9	8	3	15
	$\bar{x}\pm \sigma$	5.3 ± 0.1	5.2 ± 0.3	5.5 ± 0.2	5.6 ± 0.3	5.9 ± 0.3	5.9 ± 0.2
	Range	5.1–5.4	4.4–5.5	5.0–5.7	4.9–6.0	5.6–6.1	5.2–6.2
UPL	*n*	5	9	9	8	3	15
	$\bar{x}\pm \sigma$	3.4 ± 0.1	3.5 ± 0.1	3.9 ± 0.3	3.9 ± 0.2	4.5 ± 0.2	4.2 ± 0.2
	Range	3.2–3.6	3.4–3.7	3.6–4.4	3.5–4.2	4.4–4.7	3.8–4.6
AIL	*n*	5	7	8	8	2	13
	$\bar{x}\pm \sigma$	2.5 ± 0.1	2.6 ± 0.1	2.8 ± 0.1	2.7 ± 0.1	NA	2.9 ± 0.2
	Range	2.5–2.6	2.5–2.8	2.5–2.9	2.5–2.9	2.9–3.1	2.7–3.3
I1.P3	*n*	5	7	8	8	3	12
	$\bar{x}\pm \sigma$	7.4 ± 0.2	7.6 ± 0.4	8.1 ± 0.3	8.3 ± 0.3	9.2 ± 0.1	9.1 ± 0.3
	Range	7.1–7.6	7.0–8.0	7.7–8.8	7.7–8.7	9.2–9.3	8.6–9.5
C1.M4	*n*	5	9	9	8	3	15
	$\bar{x}\pm \sigma$	9.6 ± 0.1	9.7 ± 0.3	10.5 ± 0.3	10.7 ± 0.2	11.5 ± 0.5	11.4 ± 0.4
	Range	9.4–9.7	9.2–10.0	10.2–10.9	10.4–11	11.1–12.0	10.6–11.9
M1.M3	*n*	5	9	9	8	3	15
	$\bar{x}\pm \sigma$	4.5 ± 0.2	4.5 ± 0.1	4.8 ± 0.2	4.8 ± 0.2	5.1 ± 0.2	5.1 ± 0.1
	Range	4.3–4.7	4.4–4.7	4.6–5.0	4.4–5.2	4.8–5.2	4.9–5.3
M2W	*n*	5	9	9	8	2	15
	$\bar{x}\pm \sigma$	2.3 ± 0.1	2.2 ± 0.1	2.5 ± 0.1	2.5 ± 0.1	NA	2.5 ± 0.1
	Range	2.2–2.4	2.2–2.3	2.4–2.7	2.3–2.7	2.4–2.5	2.2–2.7
Body							
Head‐body length	*n*	3	19	31	47	28	39
	$\bar{x}\pm \sigma$	76 ± 3.8	75 ± 4.1	84 ± 6.1	88 ± 7.3	88 ± 7.1	90 ± 8.9
	Range	72–79	68–84	74–96	70–99	76–99	71–108
Tail length	*n*	3	22	29	40	27	39
	$\bar{x}\pm \sigma$	112 ± 1.2	121 ± 7.2	125 ± 8.8	129 ± 8.5	137 ± 10.4	142 ± 10.5
	Range	111–113	108–140	111–149	104–144	121–154	121–161
Brush length	*n*	3	22	29	37	27	38
	$\bar{x}\pm \sigma$	49 ± 4.7	49 ± 3.7	55 ± 5.7	56 ± 7.0	57 ± 6.0	59 ± 7.3
	Range	44–53	40–57	40–66	35–67	44–68	43–76
Hindfoot length	*n*	4	24	33	51	29	46
	$\bar{x}\pm \sigma$	25.1 ± 1.2	26.6 ± 0.8	27.1 ± 1.2	28.2 ± 1.1	30.2 ± 1.6	30.6 ± 1.6
	Range	24.2–26.6	24.7–27.4	24.2–29.3	25.6–30.5	27.8–33.3	27.6–34.3
Forearm length	*N*	4	24	33	49	29	46
	$\bar{x}\pm \sigma$	22.6 ± 0.5	24.1 ± 1.3	26.3 ± 1.4	27.1 ± 1.5	27.5 ± 1.5	27.8 ± 1.5
	Range	22.2–23.2	21.3–26.2	22.9–29.5	22.8–30.1	24.9–30.7	24.8–31.2
Ear length	*n*	4	24	32	44	29	44
	$\bar{x}\pm \sigma$	19.8 ± 1.3	20.8 ± 1.3	23.6 ± 1.6	23.9 ± 1.7	22.5 ± 2.1	24.0 ± 1.5
	Range	18.6–21.6	18.8–23.4	19.7–27.0	19.5–27.4	18.5–26.7	21.2–28.3
Head length	*n*	3	17	30	45	28	37
	$\bar{x}\pm \sigma$	27.6 ± 0.7	28.0 ± 1.3	30.4 ± 1.2	30.7 ± 1.4	32.5 ± 1.2	32.5 ± 1.6
	Range	26.8–28.2	25.8–29.9	28.6–33.5	27.5–34.0	30.1–35.1	29.9–36.1
Head width	*n*	3	17	29	44	27	39
	$\bar{x}\pm \sigma$	13.9 ± 0.4	14.5 ± 0.7	15.8 ± 1.0	15.9 ± 0.9	16.7 ± 1.3	16.8 ± 1.2
	Range	13.6–14.4	13.3–15.9	14.2–18.3	13.2–17.7	14.3–19.9	15.2–19.7

*Note:* All measurements in millimetres (mm). Measurement abbreviations as in Table 2.

Abbreviations: $\bar{x}\pm \sigma$, mean ± standard deviation; F, female; M, male; *n*, number of individuals.

**TABLE A6:** Diagnostic nucleotide differences between the *Antechinomys laniger* (kultarr) species complex within the mitochondrial 12S gene.

	89	161	165	177	212	263	384	392	447	492	493	721	942	947
*A. laniger* sensu stricto	C	C	G	A	A	A	T	C	G	A	T	T	T	C
*A. spenceri*	A	T	A	G	G	G	C	C	A	A	C	C	C	A
*A. auritus* sp. nov.	G	C	G	A	A	A	T	T	G	G	T	C	T	C

*Note:* Nucleotide positions are numbered with respect to the *Sminthopsis crassicaudata* mitochondrial genome (GenBank accession: NC_007631).
